# Insights into the genetic diversity and species distribution of *Oswaldocruzia* nematodes (Trichostrongylida: Molineidae) in Europe: apparent absence of geographic and population structuring in amphibians

**DOI:** 10.1051/parasite/2025020

**Published:** 2025-04-23

**Authors:** Kristián Gulyás, Monika Balogová, Natália Pipová, Petr Papežík, Dalibor Uhrovič, Peter Mikulíček, Tímea Brázová, Michal Benovics

**Affiliations:** 1 Department of Zoology, Faculty of Science, Pavol Jozef Šafárik University in Košice Šrobárova 2 040 01 Košice Slovakia; 2 Department of Animal Physiology, Faculty of Science, Pavol Jozef Šafárik University in Košice Šrobárova 2 040 01 Košice Slovakia; 3 Department of Zoology, Faculty of Natural Sciences, Comenius University in Bratislava Ilkovičova 6 842 15 Bratislava Slovakia; 4 Institute of Parasitology, Slovak Academy of Sciences 04001 Košice Slovakia; 5 Department of Botany and Zoology, Faculty of Science, Masaryk University Kotlářská 2 611 37 Brno Czechia; 6 Unit for Environmental Sciences and Management, North-West University Potchefstroom 2520 South Africa

**Keywords:** *Oswaldocruzia filiformis*, Amphibian Nematodes, Genetic Diversity, Phylogeography, Host-Parasite Dynamics

## Abstract

The genus *Oswaldocruzia* represents a taxonomically diverse group of nematodes with global distribution. Although *Oswaldocruzia* species are widespread and exhibit a remarkably wide host range in some species, their genetic diversity and biogeographic patterns remain poorly understood. This study investigated the genetic variability and distribution of *Oswaldocruzia* spp. in nine anuran species from the genera *Bufo*, *Bufotes*, *Pelophylax*, and *Rana* across Central Europe and the Balkans. Two species were identified: *Oswaldocruzia filiformis* and *O. ukrainae*, each exhibiting a different range of host associations. Phylogenetic analyses based on mitochondrial COI sequences revealed significant haplotype diversity in the generalist *O. filiformis*, with low geographic and host-associated genetic structuring. In contrast, *O. ukrainae*, which is closely associated with *Bufotes viridis*, exhibited only one genetic variant across all samples, highlighting its restricted genetic diversity. The findings emphasize contrasting genetic diversities among nematode parasites exhibiting different levels of host-specificity and expand the known distribution of *O. filiformis* into new regions of the Balkans. In addition, they highlight the need for additional studies on the ecological and evolutionary factors that influence the genetic diversity of parasites in amphibians.

## Abstract (fr)

Le genre *Oswaldocruzia* représente un groupe de nématodes taxonomiquement diversifié, réparti à l’échelle mondiale. Bien que les espèces d’*Oswaldocruzia* soient répandues et présentent une gamme d’hôtes remarquablement large chez certaines espèces, leur diversité génétique et leurs profils biogéographiques restent mal compris. Cette étude a examiné la variabilité génétique et la répartition des espèces d’*Oswaldocruzia* chez neuf espèces d’anoures des genres *Bufo*, *Bufotes*, *Pelophylax* et *Rana* en Europe centrale et dans les Balkans. Deux espèces ont été identifiées : *Oswaldocruzia filiformis* et *O. ukrainae*, chacune présentant une gamme différente d’associations d’hôtes. Les analyses phylogénétiques basées sur les séquences COI mitochondriales ont révélé une diversité d’haplotypes significative chez l’espèce généraliste *O. filiformis*, avec une faible structuration génétique géographique et associée à l’hôte. En revanche, *O. ukrainae*, étroitement associé à *Bufotes viridis*, ne présentait qu’une seule variante génétique dans tous les échantillons, soulignant sa diversité génétique limitée. Ces résultats mettent en évidence les diversités génétiques contrastées parmi les nématodes parasites présentant différents niveaux de spécificité d’hôte et étendent la répartition connue d’*O. filiformis* à de nouvelles régions des Balkans. De plus, ils soulignent la nécessité de mener des études complémentaires sur les facteurs écologiques et évolutifs qui influencent la diversité génétique des parasites des amphibiens.

## Introduction

Employing Wright’s concept of “isolation by distance” [115], we can expect that genetic differentiation will increase with increasing geographic distance between two groups of organisms. Geographical isolation fundamentally prevents gene flow between two groups, which has a pivotal role in the formation of demes (*e.g.*, [20, 52, 78, 80, 95]). However, distance is not the only factor affecting genetic differentiation. The other important factor is landscape isolation, strongly affecting population connectivity, which promotes population diversification even in smaller geographical regions (*e.g.*, [27, 63, 76, 101]). Therefore, the concept of “isolation by environment” was formed [108, 109], summarizing the correlation between environmental heterogeneity and spatial variation in gene flow. At first sight, the assessment of demographic structure appears to be a rather straightforward and logical task. However, a more complicated process may be recognizing demes among parasites. While geographical factors undoubtedly affect genetic structure in the parasites on a large spatial scale, we can expect that on a local scale, the structure will be more affected by ecological and environmental factors (as in vertebrates: *e.g.*, [2, 62]). In addition, the host specificity and ecology of the associated hosts also appear to be key factors, introducing an additional layer into the spatial structure of parasites [9].

Amphibians play a key role in ecosystems by regulating insect populations, serving as prey, and hosting a diverse range of parasites (trematodes, nematodes, cestodes, and acanthocephalans), contributing to helminth transmission as both intermediate and definitive hosts [16, 48]. The life cycles of these parasites are closely linked to their amphibian hosts, with potential implications for amphibian health and population stability [16]. Host mobility is a major determinant of parasite gene flow (*e.g.*, see [60]) in parasites that lack free-living stages or have free-living stages with limited dispersal. However, in parasites with free-living stages, the genetic structures of populations are primarily determined by the dispersal abilities of these stages, and also by the mode of reproduction of the parasite (sexual versus asexual) [57]. Larval dispersal and survival will play an important role in parasites with a direct life cycle, as exhibited by many nematode species.

Nematodes are among the most common parasites of amphibians (together with trematodes), particularly of the genera *Cosmocercoides* Wilkie, 1930, *Gyrinicola* Yamaguti, 1938, *Falcaustra* Lane, 1915, *Oswaldocruzia* Travassos, 1917, and *Rhabdias* Stiles & Hassall, 1905 [48]. These nematodes infect their hosts either through direct skin penetration or by the ingestion of larval stages with food [35, 36, 48]. A significant number of nematode species associated with amphibians belong to the cosmopolitan genus *Oswaldocruzia* [23], which is predominantly found in the intestines of amphibians and reptiles [31, 86, 100]. It is a taxonomically diverse group, whose representatives are often difficult to identify due to its morphological uniformity and low host specificity [23]. The genus comprises approximately 92 species [31, 51, 100, 103, 114] and is characterized by a strong speciation potential, leading to the existence of numerous closely related species [22].

The first species of the genus *Oswaldocruzia* described in the Palaearctic was *Oswaldocruzia filiformis* Goeze, 1782. This nematode is a common and widely distributed parasite of amphibians, particularly those of the genera *Bufo* and *Rana* [29, 30, 44]. It has a direct life cycle, with invasive larvae occurring on the surface of soil and vegetation, from where they usually infect host orally during its feeding [35, 44]. The variability in the size and morphology of *O. filiformis* across different host species and regions has been well-documented. The adaptability of *O. filiformis* to diverse environmental conditions is reflected by its phenotypic plasticity, enabling the species to develop distinct ecomorphs across regions with varying ecological characteristics [45]. Despite an increasing level of research on amphibian nematodes [17, 44, 50, 100, 114], molecular genetic studies on *Oswaldocruzia* remain limited [46, 47, 113], underscoring the need for further investigation.

Previous studies on the parasite fauna of amphibians in Czechia and Slovakia revealed the presence of 30 different nematode species, including seven species of *Oswaldocruzia*: *O. molgeta* Lewis, 1928, *O. goezei* Skrjabin et Schulz, 1952, *O. filiformis* (Goeze, 1782), *O. iwanitzkyi* Sudarikov, 1951, *O. ukrainae* Iwanitzky, 1928, *O. lenteixerai* Pérez Vigueras, 1939, and *O. subauricularis* (Rudolphi, 1819) [104–108]. These studies were conducted nearly half a century ago, primarily relying on the morphological identification of parasites without accounting for their genetic characterization. The aim of our study was therefore to use molecular markers to refine the distribution of species in the genus *Oswaldocruzia*, to determine their genetic variation and population-genetic structure, and to relate these to the host specificity of individual species. Because of the southward shift in amphibian species richness and endemism within Europe [93], we hypothesize that the genetic variation in *Oswaldocruzia* nematodes will only correspond weakly to the population structure of their amphibian hosts. Additionally, due to the low host specificity of *O. filiformis* [44, 45], we expect that different host species will harbor genetically closely related nematode populations.

## Material and methods

### Ethics statement

Scientific permits for material collection and processing were provided by the Directorate of Forest Management, the Ministry for the Environment and Energy of the Hellenic Republic (181012/807/28-3-2019); the National Agency of Protected Areas, the Ministry of Tourism and the Environment of the Albanian Republic (No. 480/2019), Romanian Administratia Rezervati ei Bios Ferei Delta Dunarii (no. 362/2023), and the Ministry of the Environment of the Slovak Republic (No. 2963/2013-2.2 and no. 519/2022-6.3). All live captured animals used in this study were humanely euthanized and processed in accordance with ethical guidelines and legal regulations in the respective countries where the research was conducted.

### Material collection

**Figure 1:**
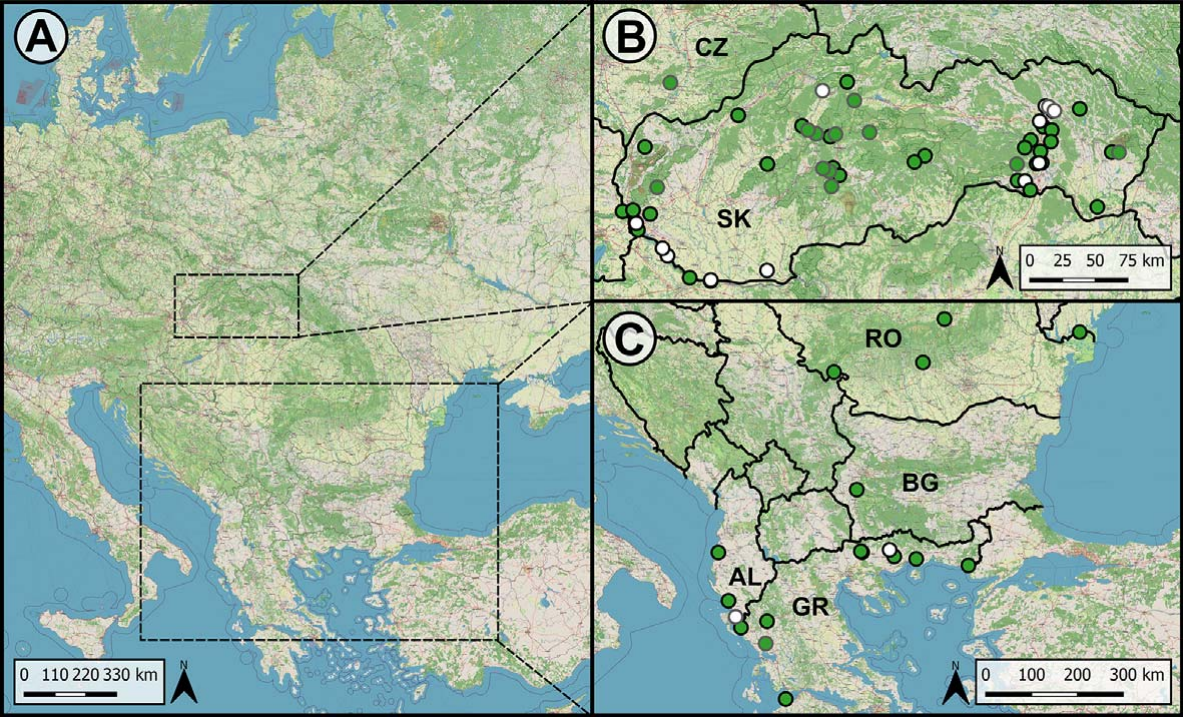
Map of sampling localities in Central Europe and the Balkans. Green markers indicate positive records of *Oswaldocruzia* spp. and white markers represent localities where no *Oswaldocruzia* spp. were recorded. Localities where fewer than three host specimens were examined are circled in grey. A = Central Europe and the Balkan peninsula; B = Central Europe (Czechia and Slovakia); C = the Balkan peninsula; AL = Albania; BG = Bulgaria; CZ = Czechia; GR = Greece; RO = Romania; SK = Slovakia.

A total of 719 frogs (either captured live or collected as cadavers and subsequently frozen) from 74 localities across the Balkans (Albania, Bulgaria, Greece, and Romania), and Central Europe (only Czechia and Slovakia) were analyzed for parasite presence (Fig. 1, Table 1, Supplementary Table S2). Nematodes were found in 11 host species: *Pelophylax ridibundus* (Pallas, 1771) (*n* = 21), *Pelophylax esculentus* (Linnaeus, 1758) (*n* = 15), *Rana temporaria* Linnaeus, 1758 (*n* = 10), *Rana dalmatina* Fitzinger, 1839 (*n* = 9), *Bufo bufo* (Linnaeus, 1758) (*n* = 224) and *Bufotes viridis* (Laurenti, 1768) (*n* = 12) and further unidentifiable *Rana* sp. (*n* = 1) in Slovakia, and *Pelophylax epeiroticus* (Schneider, Sofianidou & Kyriakopoulou-Sklavounou, 1984) (*n* = 2), *Pelophylax kurtmuelleri* (Gayda, 1940) (*n* = 7), and *Pelophylax shqipericus* (Hotz, Uzzell, Guenther, Tunner & Heppich, 1987) (*n* = 1) and further unidentified *Pelophylax* sp. (*n* = 22) in the Balkans. The identification of *Pelophylax* water frog species was based on morphological characteristics [32, 67, 72] and molecular markers, specifically sequences of the mitochondrial ND2 fragment and microsatellites. Further details regarding the ND2 and microsatellite laboratory analyses, along with the genetic identification of water frogs, can be found in studies by Plötner *et al*. [71], Hoffmann *et al*. [37], and Papežík *et al*. [67, 68]. In northeastern Greece and southern Bulgaria, where the ranges of three species that hybridize meet, water frogs have been assigned only to the genus.

**Table 1:** List of collection localities with coordinates, and number of examined frog individuals per each present species.

Country	Locality	LocID	Coordinates		Anuran species	*N*
Albania	Divjakë	DI	40.990129	19.497491	*P. kurtmuelleri*	10
					*P. shqipericus*	4
	Qazim Pali	QP	40.049669	19.841988	*P. kurtmuelleri*	9
	Xarrë	XA	39.733001	20.052892	*P. kurtmuelleri*	1
Bulgaria	Kremenik	KR	42.360640	23.074380	*Pelophylax* sp.	5
Czechia	Buchlovice	BC	49.086642	17.337341	*B. bufo*	3
Greece	Gravouna	GR	41.000261	24.670300	*Pelophylax* sp.	10
	Cheimaros	CS	41.120780	23.252460	*Pelophylax* sp.	5
	Igoumenitsa	IG	39.536593	20.203803	*P. epeiroticus*	10
					*P. kurtmuelleri*	3
	Ioannina	IO	39.688624	20.858381	*P. epeiroticus*	10
					*P. kurtmuelleri*	2
	Limanaki	LI	38.172760	21.421850	*P. epeiroticus*	10
	Lithotopos	LT	41.138280	23.219900	*Pelophylax* sp.	10
	Loutros River	LR	40.865230	26.032050	*Pelophylax* sp.	10
	Mavrolefki	MA	41.047350	24.094550	*Pelophylax* sp.	19
	Prosotsani	PR	41.169279	23.972630	*Pelophylax* sp.	10
	Zirou lake	ZL	39.240600	20.853900	*P. epeiroticus*	2
					*P. kurtmuelleri*	10
Romania	Budeasa Mică	BM	44.903817	24.846980	*P. ridibundus*	11
	Dumbrăviţa	DU	45.767861	25.458950	*P. ridibundus*	10
	Eşelniţa	ES	44.686268	22.368010	*P. ridibundus*	10
	Chilia Veche	CV	45.418647	29.276885	*P. ridibundus*	11
Slovakia	Brestov	BR	48.869871	21.348455	*B. bufo*	15
	Budča	BD	48.574399	19.069150	*B. bufo*	3
	Budiš	BU	48.874002	18.748319	*R*. *dalmatina*	4
					*Rana* sp.	1
	Cestice	CE	48.590383	21.100551	*B. viridis*	5
	Čečejovce	CC	48.598886	21.062267	*B. viridis*	5
	Číčovský stream	CR	47.764833	17.728222	*P. esculentus*	7
	Demjata	DE	49.107776	21.311242	*B. viridis*	1
	Devín	DV	48.174944	16.976694	*P. esculentus*	10
					*P. ridibundus*	11
	Dobrá Niva	DN	48.471648	19.102409	*B. bufo*	2
	Dolný Harmanec	DH	48.814759	19.049393	*R. temporaria*	6
						3
	Gbelce	GB	47.858444	18.508861	*P. ridibundus*	12
	Horná Štubňa	HS	48.823706	18.901571	*B. bufo*	1
	Hrabovo	HR	49.072325	19.274079	*B. bufo*	3
	Hronská Dubrava	HD	48.589677	19.001009	*R. temporaria*	1
	Chmeľovec	CH	49.082935	21.371885	*B. viridis*	1
	Chorvátsky stream	CHR	48.099861	17.129694	*P. ridibundus*	10
	Ivánka pri Dunaji – Lake	IJ	48.175972	17.262556	*P. ridibundus*	10
	Jasenská Dolina	JD	48.861102	19.455961	*R. dalmatina*	1
	Kolačno	KL	48.591803	18.421305	*B. bufo*	8
	Košice – KVP	KVP	48.715139	21.211511	*B. viridis*	10
	Košice – City Park	MP	48.723888	21.265415	*B. viridis*	6
	Košice – Zuzka’s park	ZP	48.718835	21.238002	*B. viridis*	5
	Kováčová	KV	48.601779	19.104349	*B. bufo*	8
	Kráľovská lúka	KRL	47.898389	17.481639	*P. ridibundus*	13
	Kusín	KS	48.813688	22.063373	*B. bufo*	2
					*R. dalmatina*	1
	Lipovec	LP	49.123771	18.933342	*B. bufo*	1
	Lomné	LO	49.106705	21.636617	*B. bufo*	16
					*R. temporaria*	4
	Malá Lodina	ML	48.875902	21.139193	*B. bufo*	15
					*R. dalmatina*	5
					*R. temporaria*	4
	Malá Vieska	MV	48.801711	21.246164	*B. bufo*	4
	Malý Draždiak	MD	48.108917	17.119361	*P. ridibundus*	5
	Modra-Harmonia	MH	48.360295	17.307702	*B. bufo*	3
	Mokrance	MK	48.593335	21.022273	*B. viridis*	5
	Môťová	MO	48.553451	19.175150	*B. bufo*	13
	Muránska Lehota	MU	48.728261	20.048167	*B. bufo*	7
	Párnica	PA	49.199106	19.184862	*R. temporaria*	8
	Perín-Chým	PCH	48.534557	21.187797	*B. Bufo*	28
					*R. dalmatina*	8
	Podhorany	PH	49.083212	21.356049	*B. viridis*	4
	Požehy	PZ	48.844820	18.814701	*B. bufo*	1
	Prešov-city square	PKP	49.006469	21.224027	*B. viridis*	5
	Prešov-Šváby	PS	48.971488	21.266167	*B. viridis*	5
	Rad site Hrušov	RH	48.435857	21.861214	*B. viridis*	5
	Rudník Lake	SR	48.702849	21.008252	*B. bufo*	1
	Rusovce	RS	48.057444	17.153111	*P. esculentus*	10
	Ružín	RU	48.822152	21.081126	*B. bufo*	22
	Sigord	SI	48.950347	21.353007	*B. bufo*	43
					*R. temporaria*	6
					*R. dalmatina*	1
	Staré Hory	SH	48.831426	19.102745	*R. temporaria*	1
					*Rana* sp.	1
	Šaštín-Stráže	SS	48.632139	17.139361	*P. esculentus*	10
	Šulianske Lake	SJ	47.946167	17.423222	*P. ridibundus*	10
	Tisovec	TS	48.68096	19.943049	*B. bufo*	14
	Veľký Lél – Island	VLO	47.754861	17.944639	*P. esculentus*	11
	Veľký Lél – Lake	VLS	47.759833	17.944722	*P. esculentus*	10
					*P. ridibundus*	10
	Vinné	VI	48.816976	21.989034	*B. bufo*	43
	Vyšný Slivník	VS	49.112080	21.275219	*B. viridis*	4
	Zamarovce	ZA	48.909715	18.074896	*B. bufo*	13
	Železná studnička	ZS	48.190172	17.082313	*B. bufo*	13

*Note*: Respective locality IDs (LocID) correspond to those used in relevant tables, and Results and Discussion sections.

Prior to the examination, frozen frogs were thawed, and live frogs were euthanized using clove oil extract and subsequently sacrificed by cutting the spinal cord. The internal organs were then placed in a saline solution and examined under a stereomicroscope. All the helminths, including nematodes, were removed from the intestines, killed with hot saline solution (0.85% NaCl) (excluding those already killed by freezing), and preserved in 70% or 96% ethanol for subsequent molecular analyses. A total of 2,563 *Oswaldocruzia* nematodes were extracted from the frogs’ small intestines. Basic quantitative descriptors of the parasite populations, such as prevalence, mean abundance, and the minimum and maximum intensities of infection, were calculated for each parasite species, as outlined by Bush *et al*. [15]. Prevalence, defined as the percentage of frogs infected by a specific parasite species, and mean abundance, defined as the average number of parasite specimens per individual host (including both infected and uninfected hosts), were calculated. To interpret the quantitative data, a 95% confidence interval was calculated for the mean abundance, following the recommendation by Rózsa *et al.* [81]. Due to the relatively small sample size of hosts per population, the bias-corrected and accelerated bootstrap (BCa) method was employed, using QPweb [77], to calculate the confidence interval for mean abundances. Selected non-damaged specimens, representing paragenophores, were deposited in the Helminthological Collection of the Institute of Parasitology of the Czech Academy of Sciences (IPCAS), Czechia, under the accession number IPCAS N-1291.

### Genomic DNA extraction, amplification, sequencing

Prior to DNA extraction, the nematodes were identified to genus level by their localization in the intestine of the hosts and general morphology. Then, they were randomly selected from each locality for DNA extraction. Total nematode genomic DNA was extracted using either a DNeasy Blood & Tissue Kit (QIAGEN, Hilden, Germany) or NucleoSpin^®^ Tissue kit (Macherey-Nagel, Düren, Germany), following the respective manufacturer’s protocol. The fragment of the mitochondrial cytochrome oxidase c subunit I gene (COI) was amplified for each *Oswaldocruzia* specimen – from at least three host specimens from each host population or all of them, if fewer specimens per population were available. For amplification, the primers JB3 (5′-TTTTTTGGGCATCCTGAGGTTTAT-3′) and JB4.5 (5′-TAAAGAAAGAACATAATGAAAATG-3′) [11] were used, and polymerase chain reactions (PCR) were carried out in a total volume of 15 μL containing 1U of DreamTaq DNA polymerase (Thermo Fisher Scientific, Waltham, MA, USA), 1× Taq Buffer, 1.5 mM MgCl_2_, 300 μM of each dNTP, 0.5 μM of each primer, 2 μL of DNA template (corresponding to 20 ng/μL) and nuclease-free water. The PCR conditions followed the protocol optimized by Kirillova *et al.* [44]. PCR products were detected by electrophoresis in 1.5% agarose gels stained with GoodView (SBS Genetech, Beijing, China). Amplified products were purified using EPPiC Fast (A&A Biotechnology, Gdansk, Poland), following the manufacturer’s protocol. Sequencing was performed in both directions using the PCR primers. Commercial services provided by Macrogen Europe (the Netherlands) were used for sequencing.

### Sequence dataset assembly and phylogenetic analyses

Prior to sequence alignment, GenBank was screened to obtain homologous COI sequences of *Oswaldocruzia* spp. A total of 21 *O. filiformis* and 6 *O. ukrainae* sequences originating from Russia, and one *Oswaldocruzia* sp. sequence from a specimen from Italy were retrieved from GenBank (Supplementary Table S1). To assess whether all conspecific sequences formed a monophyletic group, the sequence alignment was built using the Fast Fourier transform algorithm implemented in MAFFT software [43] and included all available and newly generated *Oswaldocruzia* COI sequences; two *Ancylostoma* sequences [*A. ceylanicum* (MW549613) and *A. tubaeforme* (NC_034289)] were used as an outgroup for rooting of the phylogram. The alignment was then manually trimmed to unify the length of all sequences, and translated into amino acids to avoid any signal misreads using Universal Invertebrate Mitochondrial Code (transl_table=5). Identical sequences were then removed for phylogenetic analyses. The sequence data were treated as codon partitioned, and a GTR model was selected independently for each position within the codon, including both a gamma distribution and the proportion of invariable sites. Phylogenetic trees were constructed using Bayesian inference (BI) and maximum likelihood (ML) approaches in MrBayes v. 3.2. [79] and RAxML v. 8.1.12 [98, 99], respectively. BI analysis used the Metropolis-coupled Markov chain Monte Carlo algorithm with two parallel runs of one cold and five hot chains and was run for 10^7^ generations, sampling trees every 100 generations. The initial 30% of all saved trees were discarded as “burn-in” after checking that the standard deviation split frequency fell below 0.01. The convergence of the runs and the parameters of individual runs were checked using Tracer v. 1.7.1 [75]. Posterior probabilities for each tree node were calculated as the frequency of samples recovering a given clade. The clade bootstrap support for ML trees was assessed by simulating 10^3^ pseudoreplicates.

A subsequent dataset was built from all available *O. filiformis* sequences, including the newly generated ones, and was used to assess intraspecific genetic variability on the wide geographical range level. The level of DNA polymorphism in COI sequences, i.e., haplotype diversity (Hd), nucleotide diversity (π), the number of unique haplotypes, and the number of variable sites, was assessed using DnaSP 5 [55]. To examine genetic diversity, the following statistical analyses were performed in R v. 4.1.3 [74]. A pairwise matrix of uncorrected *p*-distances was generated using the dist.dna() function from the ape package [69]. This matrix was used as input for principal coordinates analysis (PCoA) conducted with the vegan package [65] to visualize genetic diversity in multivariate space. Based on the position of each individual in the multivariate space, 95% confidence interval (CI) ellipses were drawn around the individuals to facilitate easier interpretation. The results were visualized using the ggplot2 package [112]. Uncorrected *p-*distance calculations and PCoA were performed in R v. 4.1.3.

Lastly, a median-joining haplotype network constructed in PopART [53] was used to assess the population genetic structure of *O. filiformis* based on COI haplotypes from individuals in Slovakia with respect to individual major river basins and host associations. The dataset included all *O. filiformis* sequences originating from individuals from the Central European amphibians. All sequences used in the present study, including those newly generated, are presented in Supplementary Table S1. New sequences were deposited in GenBank with accession numbers PV168566–PV168633.

## Results

### Diversity and distribution of *Oswaldocruzia* spp.

**Supplementary Figure S1:**
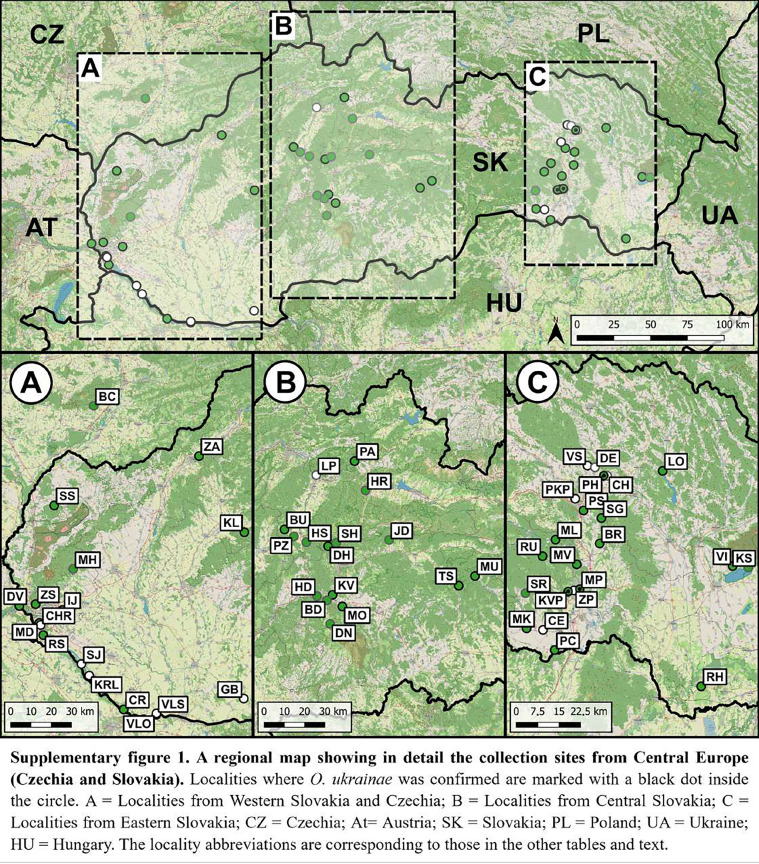
Regional map showing in detail the collection sites from Central Europe (Czechia and Slovakia). Localities where *O. ukrainae* was confirmed are marked with a black dot inside the circle. A = Localities from Western Slovakia and Czechia; B = Localities from Central Slovakia; C = Localities from Eastern Slovakia; CZ = Czechia; AT = Austria; SK = Slovakia; PL = Poland; UA = Ukraine; HU = Hungary. The locality abbreviations correspond to those in the other tables and text.

**Supplementary Figure S2:**
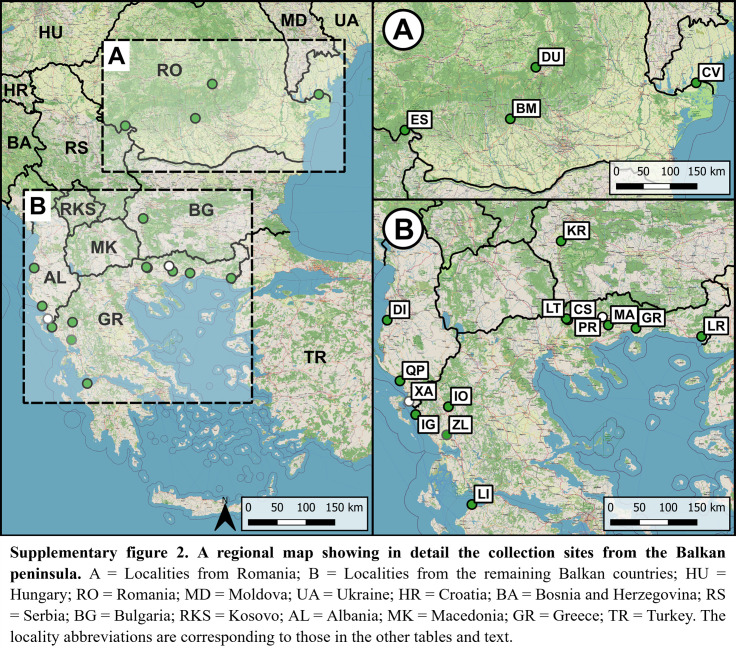
Regional map showing in detail the collection sites from the Balkan peninsula. A = Localities from Romania; B = Localities from the remaining Balkan countries; HU = Hungary; RO = Romania; MD = Moldova; UA = Ukraine; HR = Croatia; BA = Bosnia and Herzegovina; RS = Serbia; BG = Bulgaria; RKS = Kosovo; AL = Albania; MK = Macedonia; GR = Greece; TR = Türkiye. The locality abbreviations correspond to those in the other tables and text.

A total of 330 anurans (out of 719) were infected by nematodes of the genus *Oswaldocruzia*, out of which we identified two species: *Oswaldocruzia filiformis* and *O. ukrainae. Oswaldocruzia ukrainae* was found only in *B. viridis* from three Slovak localities (KVP, MP, PH) (see Supplementary Figs. 1 and 2 for localities with abbreviated IDs). We confirmed the occurrences of *Oswaldocruzia filiformis* specimens both in Central Europe (Czechia and Slovakia), and the Balkans (Albania, Bulgaria, Greece, and Romania) (Fig. 1, Figs. S1, S2). By excluding the negative localities, the prevalence of *Oswaldocruzia* samples in the studied frog populations varied from 9.1% (VLO, Slovakia – *P. esculentus*) to 100% [BR; BU (*Rana* sp. only); HS; HD; JD; KS; MV; MH; PZ; SH (*R. temporaria* only); SR – each from Slovakia and IO (*P. kurtmuelleri* only) – from Greece] (Table 2, abbreviations for localities are in Table 1). From all the populations with a 100% prevalence of *Oswaldocruzia* specimens, only the BR, MV, and MH populations had more than one host sample examined. From the localities where more than one host individual was examined, the highest mean abundance (21) and intensity of infection (7–48) was recorded among *Bufo bufo* at MH (Slovakia).

**Table 2:** Epidemiologic characteristics of the *Oswaldocruzia* parasites calculated for each host population.

Country	LocID	Species	*N*	P	A	95% confidence interval for mean abundance	I
Albania	DI	*P. kurtmuelleri*	10	0.0%	–	–	–
		*P. shqipericus*	4	25.0%	0.25	0–0.50	1
	QP	*P. kurtmuelleri*	9	11.0%	0.33	0–1.00	3
	XA	*P. kurtmuelleri*	1	0.0%	–	–	–
Bulgaria	KR	*Pelophylax* sp.	5	20.0%	2.00	0–4.00	10
Czechia	BC	*B. bufo*	3	66.7%	12.67	0–20.30	15–23
Greece	GR	*Pelophylax* sp.	10	10.0%	0.10	0–0.30	1
	CS	*Pelophylax* sp.	5	40.0%	0.60	0–1.20	1–2
	IG	*P. epeiroticus*	10	0.0%	–	–	–
		*P. kurtmuelleri*	3	33.3%	0.67	0–1.33	2
	IO	*P. epeiroticus*	10	10.0%	0.10	0–0.30	1
		*P. kurtmuelleri*	2	100.0%	15.50	7–15.50	7–24
	LI	*P. epeiroticus*	10	10.0%	0.20	0–0.60	2
	LT	*Pelophylax* sp.	10	50.0%	1.70	0.50–3.30	1–6
	LR	*Pelophylax* sp.	10	70.0%	1.90	0.90–2.80	1–4
	MA	*Pelophylax* sp.	19	31.6%	0.90	0.32–1.95	1–6
	PR	*Pelophylax* sp.	10	0.0%	–	–	–
	ZL	*P. epeiroticus*	2	0.0%	–	–	–
		*P. kurtmuelleri*	10	30.0%	0.60	0–1.30	1–3
Romania	BM	*P. ridibundus*	11	18.2%	0.27	0–0.73	1–2
	DU	*P. ridibundus*	10	40.0%	1.00	0.20–2.79	1–6
	ES	*P. ridibundus*	10	60.0%	2.90	0.09–6.60	1–14
	CV	*P. ridibundus*	11	27.3%	0.27	0–0.46	1
Slovakia	BR	*B. bufo*	15	100.0%	11.50	8.2–15.7	4–27
	BD	*B. bufo*	3	66.7%	1.30	0–2.33	1–3
	BU	*R*. *dalmatina*	4	50.0%	1.75	0–3.00	3–4
		*Rana* sp.	1	100.0%	14.00	–	14
	CE	*B. viridis*	5	0.0%	–	–	–
	CC	*B. viridis*	5	20.0%	0.20	0–0.40	1
	CR	*P. esculentus*	7	28.6%	1.29	0–4.71	1–8
	DE	*B. viridis*	1	0.0%	–	–	–
	DV	*P. esculentus*	10	30.0%	0.40	0–0.80	1–2
		*P. ridibundus*	11	54.5%	1.45	0.55–2.36	1–4
	DH	*R. temporaria*	6	50.0%	1.50	0.17–3.33	1–5
		*R. dalmatina*	3	33.3%	0.33	0–0.67	1
	GB	*P. ridibundus*	12	0.0%	–	–	–
	HS	*B. bufo*	1	100.0%	9.00	–	9
	HR	*B. bufo*	3	66.7%	6.33	0–12.00	2–17
	HD	*R. temporaria*	1	100.0%	10.00	–	10
	CH	*B. viridis*	1	0.0%	–	–	–
	CHR	*P. ridibundus*	10	0.0%	–	–	–
	IJ	*P. ridibundus*	10	40.0%	1.40	0.20–4.70	1–11
	JD	*R. dalmatina*	1	100.0%	23.00	–	23
	KL	*B. bufo*	8	62.5%	7.63	2.62–13.80	2–19
	KVP	*B. viridis*	10	40.0%	0.90	0.20–1.90	1–4
	MP	*B. viridis*	6	66.7%	9.00	1–22.80	1–30
	ZP	*B. viridis*	5	0.0%	0.00	–	–
	KV	*B. bufo*	8	75.0%	5.00	1.38–13.60	1–24
	KRL	*P. ridibundus*	13	0.0%	–	–	–
	KS	*B. bufo*	2	100.0%	11.00	45601.00	5–17
		*R. dalmatina*	1	100.0%	1.00	–	1
	LP	*B. bufo*	1	0.0%	–	–	–
	LO	*B. bufo*	16	68.8%	2.50	1.38–4.06	1–9
		*R. temporaria*	4	0.0%	–	–	–
	ML	*B. bufo*	15	86.7%	4.87	2.73–8.13	1–17
		*R. dalmatina*	5	20.0%	0.40	0–0.80	2
		*R. temporaria*	4	0.0%	–	–	–
	MV	*B. bufo*	4	100.0%	7.00	5–7.75	5–8
	MD	*P. ridibundus*	5	40.0%	7.40	0–22.00	1–36
	MH	*B. bufo*	3	100.0%	21.00	7–34.70	7–48
	MK	*B. viridis*	5	0.0%	–	–	–
	MO	*B. bufo*	13	69.2%	6.08	3.23–11.60	2–25
	MU	*B. bufo*	7	42.9%	1.86	0.14–4.86	1–9
	PA	*R. temporaria*	8	25.0%	0.50	0–1.38	1–3
	PCH	*B. Bufo*	28	82.1%	5.89	4.11–9.51	1–32
		*R. dalmatina*	8	37.5%	0.75	0.125–1.62	1–3
	PH	*B. viridis*	4	25.0%	1.00	0–2.00	4
	PZ	*B. bufo*	1	100.0%	2.00	–	2
	PKP	*B. viridis*	5	0.0%	–	–	–
	PS	*B. viridis*	5	20.0%	0.20	0–0.40	1
	RH	*B. viridis*	5	20.0%	0.40	0–0.80	2
	RS	*P. esculentus*	10	30.0%	0.80	0.10–2.60	1–6
	RU	*B. bufo*	22	86.4%	9.05	5.77–13.70	1–36
	SI	*B. bufo*	43	97.7%	14.30	11.7–17.9	1–49
		*R. temporaria*	6	50.0%	3.50	0.67–6.67	4–9
		*R. dalmatina*	1	0.0%	–	–	–
	SH	*R. temporaria*	1	100.0%	18.00	–	18
		*Rana* sp.	1	0.0%	–	–	–
	SS	*P. esculentus*	10	60.0%	2.20	0.75–4.60	1–9
	SR	*B. bufo*	1	100.0%	2.00	–	2
	SJ	*P. ridibundus*	10	0.0%	–	–	–
	TS	*B. bufo*	14	71.4%	5.71	2.53–10.70	1–20
	VLO	*P. esculentus*	11	9.1%	0.27	0–0.82	3
	VLS	*P. esculentus*	10	0.0%	–	–	–
		*P. ridibundus*	10	0.0%	–	–	–
	VI	*B. bufo*	43	76.7%	5.49	3.58–8.77	1–43
	DN	*B. bufo*	2	50.0%	0.50	0–0.50	1
	VS	*B. viridis*	4	0.0%	–	–	–
	ZA	*B. bufo*	13	76.9%	4.77	2.77–7.77	2–15
	ZS	*B. bufo*	13	46.2%	7.69	2.69–16.10	1–35

*N*, number of processed host individuals; P, prevalence; A, mean abundance (with a 95% confidence interval); I, range of intensity of infection; absence of data is marked by dashes (–).

### Phylogenetic relationships and intraspecific genetic variability of *Oswaldocruzia* spp.

**Figure 2:**
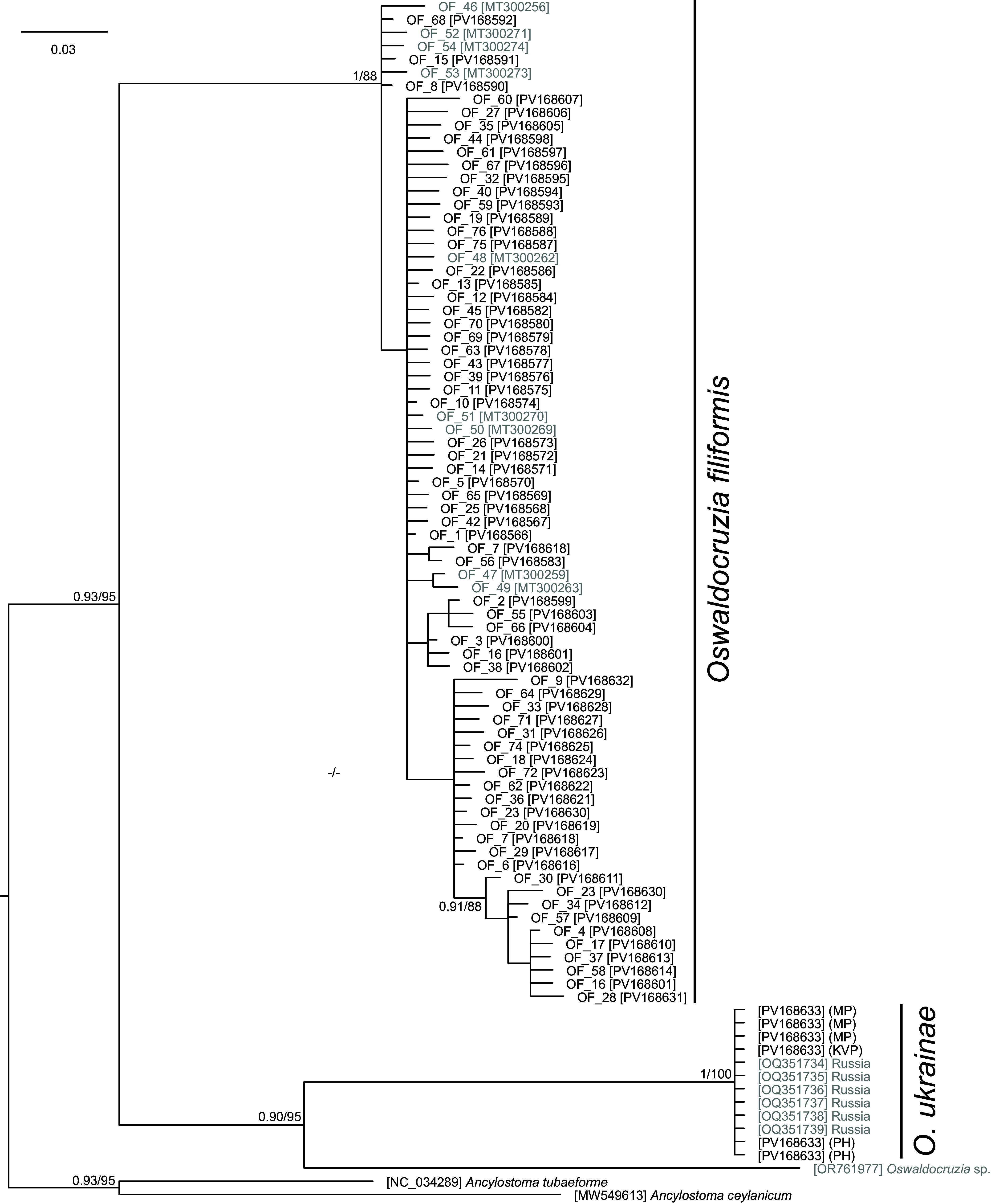
Phylogenetic tree of 90 COI sequences of three *Oswaldocruzia* species reconstructed by Bayesian inference. The tree is based on a 370 bp-long alignment and rooted using *Ancylostoma tubaeforme* and *A. ceylanicum* as the outgroup. Each *Oswaldocruzia filiformis* represents a unique haplotype. Haplotype numbers correspond to those in Figure 4 and Supplementary Table S1. Values at the nodes indicate posterior probabilities (>70) from the Bayesian inference, and bootstrap values (>50) from the maximum likelihood analysis. Lower values are shown as dashes (–). The length of branches represents the number of substitutions per site. The sequences retrieved from GenBank are greyed.

Based on the quality and lengths of the sequences, a total of 174 newly generated *Oswaldocruzia* spp. COI sequences were selected for subsequent phylogenetic analyses. A total of 76 unique COI haplotypes were recognized in *O. filiformis*. All *O. ukrainae* specimens carried the same genetic variant of the COI gene. Identical *O. filiformis* sequences were removed from the dataset for phylogenetic tree reconciliation (i.e., each haplotype is represented only by a single sequence, see Supplementary Table S1 for selected sequences), and the final alignment was built on 91 sequences (also including two *Ancylostoma* orthologous sequences as an outgroup for rooting the phylogenetic tree and 28 *Oswaldocruzia* sequences retrieved from GenBank) and spanned 370 unambiguously aligned nucleotide positions. Both phylogenetic analyses (BI and ML) generated trees with congruent topologies, and therefore only the BI tree is presented with posterior probabilities and bootstrap support values (Fig. 2). The phylogenetic analyses congruently confirmed the monophyly of all conspecific sequences. The dataset also included the sequence of *Oswaldocruzia* sp. from *Elaphe quatuorlineata* from Italy, which was in a sister position to the cluster encompassing all *O. ukrainae* sequences.

**Figure 3:**
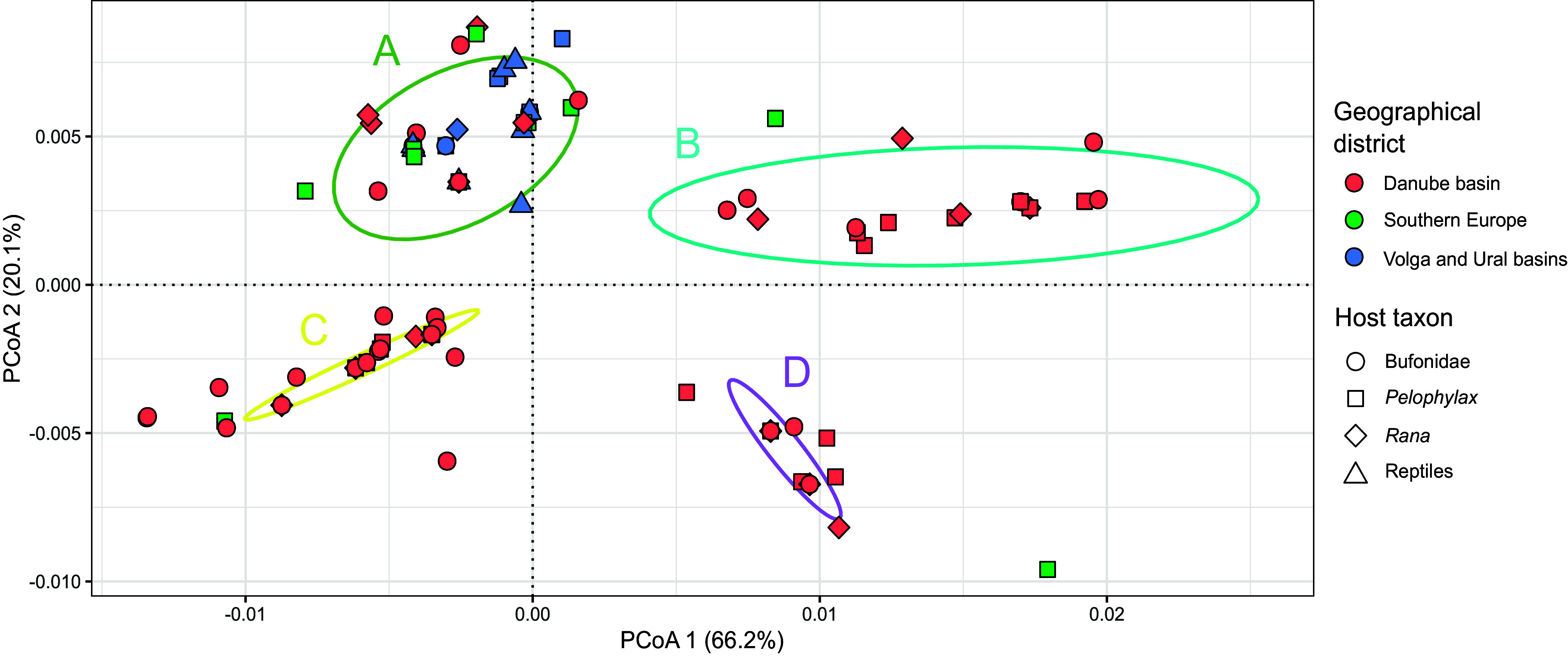
Principal coordinates analysis (PCoA) showing genetic diversity among the analysed *Oswaldocruzia filiformis* COI sequences obtained in Central Europe and the Balkans. The colors and shapes of the marks are associated with the major geographical district and host taxon, respectively.

**Supplementary Figure S3:**
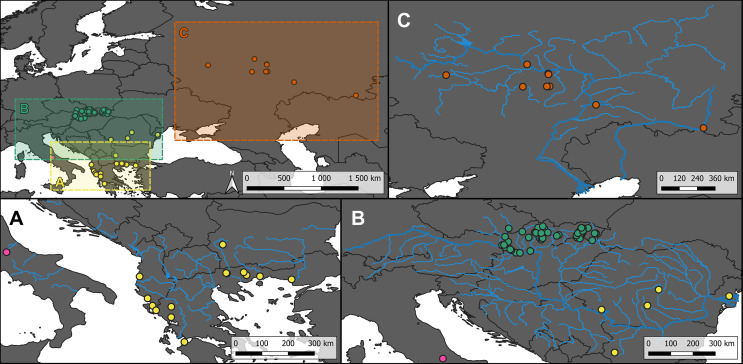
Defined river basins used in the PcoA analyses. Dots on the map represent all sequences used in the PcoA analyses obtained during this study or from the GenBank database. Green dots represent localities with novel data from Central Europe, yellow dots represent localities with novel data from the Balkan peninsula, pink dots represent the Italian locality of *Oswaldocruzia* sp., for which sequence data were obtained from the GenBank database from Italy, and brown dots represent the Russian localities of *Oswaldocruzia* spp., for which sequence data were retrieved from GenBank. A = South-eastern European River basins; B = Danube River basin; C = Volga and Ural River basins.

Within 76 unique haplotypes recognized among 189 *O. filiformis* COI sequences (including those retrieved from GenBank), 53 polymorphic sites were identified. Out of all haplotypes, 19 were recorded from more than a single *Oswaldocruzia* individual. These were also almost exclusively present in more than one locality. The uncorrected *p*-distances between haplotypes ranged between 0.3 and 3.5%. The overall haplotype diversity (Hd) was 0.954, and nucleotide diversity (π) reached 1.1%. The translated sequence alignment spanned 123 amino acid positions and only three sites were identified as polymorphic, *i.e.*, the majority of the nucleotide substitutions were recognized as synonymic. The results of PCoA divided all haplotypes into four relatively well-separated, but artificially defined clusters (Fig. 3). No apparent pattern was observed based on host associations. A weak degree of geographic structuring was recognized among the four clusters. Cluster A encompassed the majority of haplotypes carried by *O. filiformis* individuals from the Volga/ and Ural basins and southern Balkans. The majority of haplotypes within cluster B were from individuals from the Danube basin; however, this cluster also encompassed the single haplotype from the southern Balkans [OF_9, carried by a single individual from the LR locality, and also by an individual in BM (Romania)]. Cluster C again encompassed mainly haplotypes from the Danube basin. However, it also included the widespread haplotype OF_1, which was recognized among individuals in the Danube basin (Slovakia – various localities), the Southern Balkans (Albania – QP, Bulgaria – KR, and Greece – GR, LR, and AR), and the Volga basin (Russia – Uzola floodplains), and haplotype OF_10 from the Volga and from the Danube basin. This cluster also included the unique haplotype OF_40, found in only one individual of *O. filiformis* at LR. The last cluster, labelled D, contained only haplotypes from *O. filiformis* specimens in the Danube basin, and three unique haplotypes from Greece, specifically from IO and LR. Notably, the unique haplotype OF_28, found in an *O. filiformis* specimen from Ioannina Lake (Greece), was distinct from all four clusters on the first two dimensions. The river basins and the localities related to each basin are shown in Supplementary Figure S3.

### Genetic variability and distribution of *O. filiformis* in Central Europe

**Figure 4:**
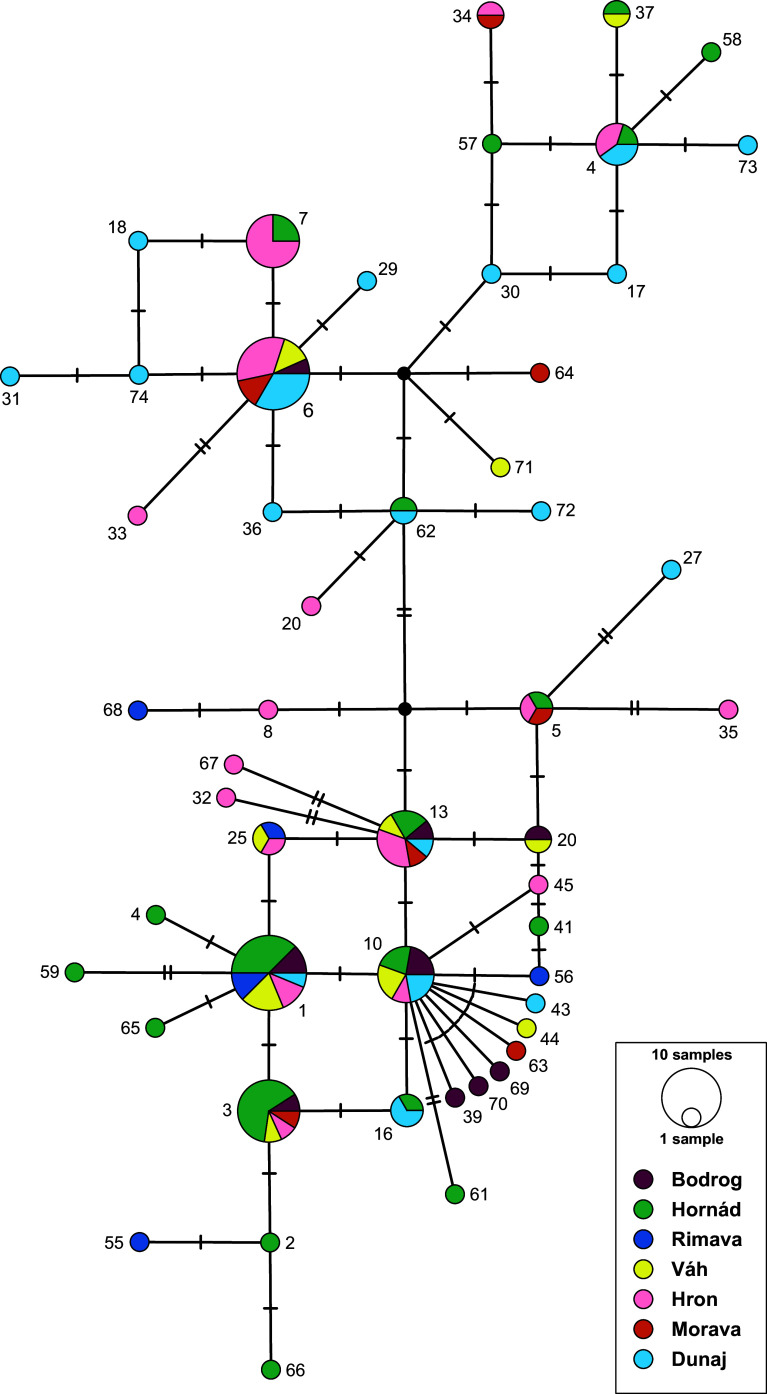
Population-genetic structure of *Oswaldocruzia filiformis* found in Slovak populations of frogs, based on COI haplotypes presented as a median-joining haplotype network. The sizes of the circles in the network are proportional to the relative frequencies of the haplotypes; small black circles represent missing haplotypes. The vertical lines represent the number of substitutions between individual haplotypes. Different colors represent major river basins according to the legend. The haplotype numbers correspond to those in Figure 1 and Supplementary Table S1.

**Figure 5:**
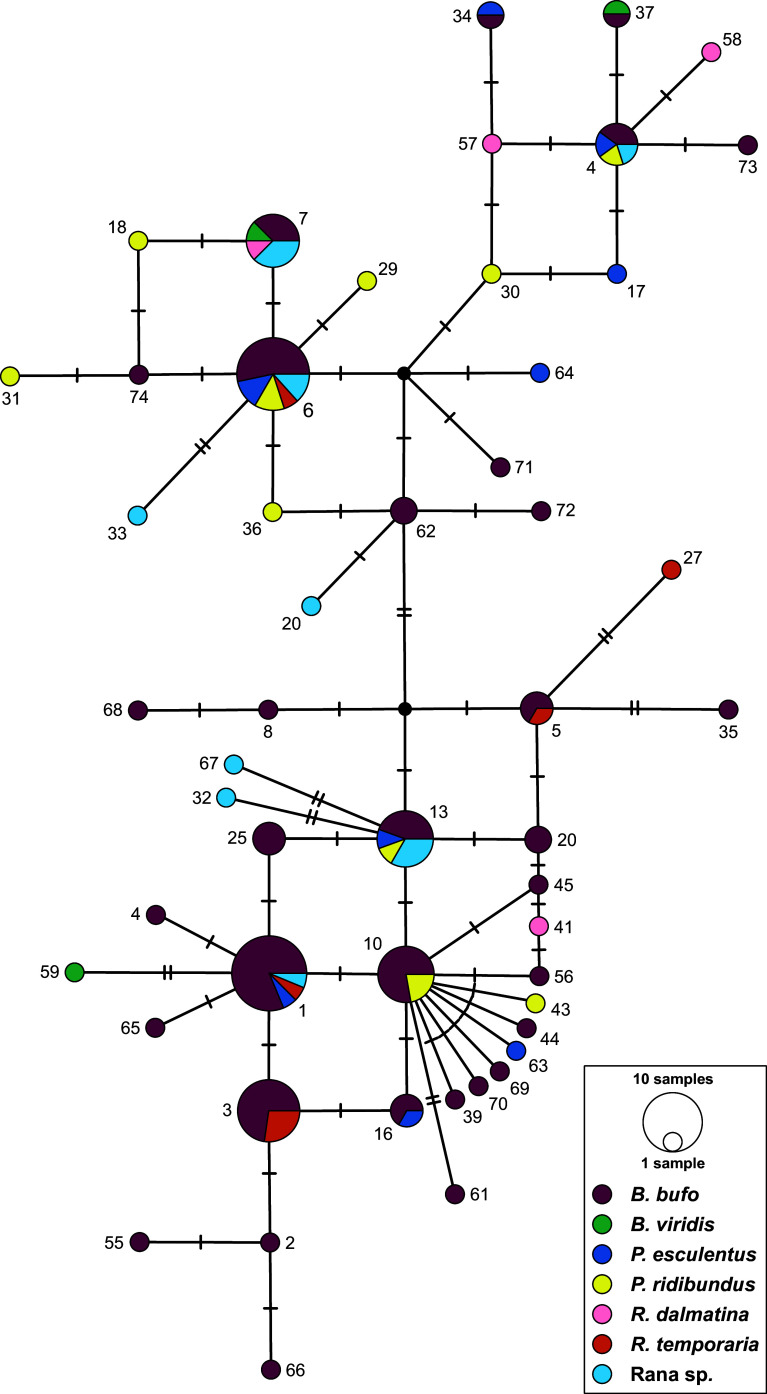
Population-genetic structure of *Oswaldocruzia filiformis* found in Slovak populations of frogs, based on COI haplotypes presented as a median-joining haplotype network. The sizes of the circles in the network are proportional to the relative frequencies of the haplotypes; small black circles represent missing haplotypes. The vertical lines represent the number of substitutions between individual haplotypes. Different colors represent specific host taxa for *O. filiformis* according to the legend. The haplotype numbers correspond to those in Figure 1 and Supplementary Table S1.

The haplotype network, with trait groups based on the major river basins in Central Europe (Fig. 4), showed no associations across the distribution of the haplotypes in Central Europe with local river basins. *Oswaldocruzia filiformis* specimens from Central Europe carried 51 COI haplotypes (out of 76 in total), out of which 46 were not recognized among the individuals from other countries. The highest haplotype diversity was recognized among specimens collected from *B. bufo* (Fig. 5), with 22 being unique for the *O. filiformis* specimens from this host. Six haplotypes were unique for the individuals from *P. ridibundus*. A total of 14 haplotypes were identified in multiple *O. filiformis* specimens (i.e., OF_1, OF_3, OF_4, OF_5, OF_6, OF_7, OF_10, OF_13, OF_16, OF_20 OF_25, OF_34, OF_37, OF_62). The most common haplotypes among *O. filiformis* in Central Europe were OF_1 (recognized in 16 specimens) and OF_6 (in 15 specimens). The overall haplotype diversity of *O. filiformis* in Central Europe was 0.950, and nucleotide diversity reached a value of 1.2%.

## Discussion

While parasites of economically important animals, such as fish and livestock, as well as those affecting humans are being extensively studied [1, 4, 83, 121], parasites of amphibians are receiving comparatively less attention. Although their direct impact on human health is limited, understanding host-parasite relationships is essential for recognizing their ecological roles in, and contributions to overall ecosystem dynamics. This study investigated the genetic variability of *Oswaldocruzia* species collected from various amphibian hosts across several Central European and Balkan countries and compared these findings with existing COI sequences of *Oswaldocruzia* obtained by Kirillova *et al.* [44] from European Russia and by Mendoza-Roldan *et al.* [58] from Italy.

### The occurrence of *Oswaldocruzia* spp. in the Palearctic

The genus *Oswaldocruzia* has been reported worldwide, with studies documenting its occurrence across various continents such as South America [31, 96, 114], Central America [13, 14], North America [34], Europe [23, 97, 106], Africa [5], Australia [6], and Asia [25, 56, 119]. Here, we report the occurrence of the genus *Oswaldocruzia* in nine anuran species from the genera *Pelophylax*, *Rana*, *Bufo*, and *Bufotes* across two Central European countries (Czechia and Slovakia) and the Balkan Peninsula, including Albania, Romania, Bulgaria, and Greece (Table 1). Two species of *Oswaldocruzia* were identified: *O. filiformis* and *O. ukrainae*. The first species, *O. filiformis*, is considered to have a widespread distribution and broad host range [29, 44]. A previous study in Bulgaria [12] recorded *O. filiformis* in various anurans, such as *Bombina bombina*, *Bombina variegata*, *B. bufo*, *B. viridis*, *Hyla arborea*, *Rana graeca, R. temporaria*, and *R. dalmatina*. In Türkiye, its occurrence was documented in *B. bufo* [24], *B. viridis*, and *Pelophylax* sp. [24, 42], *Hyla arborea* [42], as well as in *Rana macrocnemis* [117] and *Pelodytes caucasicus* [118]. Additionally, *O. filiformis* has been identified in caudate amphibians such as *Salamandra salamandra* from Bulgaria [12], *Lissotriton vulgaris* from Belarus [92] and Germany [94], and *Ommamotriton vittatus* from Türkiye [120]. Reports from reptiles include occurrences in *Lacerta trilineata* (Türkiye [116]), *Lacerta viridis* (Czechia [10]), *Zootoca vivipara* (Spain [85]; Belarus [90]), *Anguis fragilis*, *Natrix natrix*, and *Vipera berus* (Belarus [91]). Other European reports span Russia [64], Poland [66], France [97], Ireland [30], Hungary [36], Ukraine [50], Austria [87], and Czechia and Slovakia [106]. Our study expands the known distribution of *O. filiformis* to other Balkan countries, such as Romania, Greece, and Albania, and increases its host range to 30 amphibian and reptile species. Previous studies on *O. ukrainae* indicate its occurrence within a limited range of hosts, including *Bombina bombina* (Linnaeus, 1758), *B. variegata* (Linnaeus, 1758), *B. bufo*, *B. viridis*, and *Rana arvalis* [46], with the highest number of reports observed in *B. viridis* [5, 106], which may suggest a potential host association with *B. viridis*. Our results further support this possibility, as the only host species of *O. ukrainae* in this study was *B. viridis*. Nevertheless, in certain populations, *B. viridis* was also found to be infected with *O. filiformis* (Slovak localities PS and KVP) in this study. However, no cases of co-infection with other *Oswaldocruzia* species were identified. This finding is not unique, as previous studies [106, 116] also reported *B. viridis* as a host of *O. filiformis*.

### Epidemiology of *O. filiformis* in the Palearctic

Chikhlyaev *et al.* [18] and Griffin [30] documented fluctuations in the prevalence and abundance of *O. filiformis* in bufonid and ranid frogs across studied localities in Russia and Ireland, respectively. These observations align with our findings, where *O. filiformis* in *B. bufo* hosts showed variability in prevalence and abundance across the studied localities. Kuzmin *et al.* [50] also reported a fluctuating trend in *Oswaldocruzia* infections across different *Pelophylax* species in northern Ukraine. They found a substantially lower prevalence and abundance of *Oswaldocruzia* spp. in *P. esculentus* compared to the investigated populations of *P. ridibundus*. However, this does not align with our observations, where the two *Pelophylax* species exhibited similar epidemiological indices across the investigated populations. These contrasting and highly variable results suggest a complex infection landscape for *Oswaldocruzia* spp. within different anuran populations and localities. This variability may be associated with factors such as the ecology of host species, host population densities, and parasite transmission dynamics, or may reflect the sampling effort, which varies among studies (*e.g.*, [70, 73, 82]).

### Population-genetic structure of *Oswaldocruzia* spp.

To the best of our knowledge, only three studies [46, 47, 94] have specifically focused on the genetic variability of *Oswaldocruzia* species in Europe so far. Sinsch *et al.* [94] identified *O. filiformis* from *Lissotriton vulgaris* on the basis of morphology and DNA sequences. Initially, Kirillova *et al.* [44] investigated the genetic variability of *O. filiformis* in correlation with morphological characteristics across the Volga and Ural basins. Later, Kirillova *et al.* [46] analyzed *O. ukrainae* COI sequences in *B. viridis.* By analyzing 174 newly-obtained COI sequences, we confirmed the presence of two *Oswaldocruzia* species in this study. Congruently with Kirillova *et al.* [46], all *O. ukrainae* specimens possessed a single COI variant. In contrast to *O. ukrainae*, our analyses revealed substantial genetic variability within *O. filiformis*, as 76 different COI haplotypes were recognized. *Oswaldocruzia filiformis* was the first species of the genus *Oswaldocruzia* described in the Palaearctic. This nematode is a common and widely distributed parasite of amphibians, particularly those of the genera *Bufo* and *Rana* [29, 30, 44]. The contrasting level of genetic variability is not unusual among parasites (*e.g.*, [3, 26, 33, 41, 59, 61, 102]. Host-specific nematodes are limited to a narrower range of hosts, which reduces opportunities for gene flow among populations. This can lead to a smaller effective population size and a decrease in genetic diversity over time. Moreover, due to host specialization, specialist species tend to evolve traits or genetic forms tightly suited to their host, potentially limiting their ability to adapt to other environments or hosts and further reinforce certain traits within the populations. On the other hand, generalist nematodes exploit multiple host species and a broader range of ecological niches, further promoting higher genetic variability as a result of broader population dynamics and opportunities to adapt to more diverse environments (see [19, 38, 60]). In the generalist *O. filiformis*, we assumed that the haplotype variation would be associated with either host species (similarly as in Shaw *et al.* [89] and Shaw *et al.* [88]) or geographical distribution (as in [54]); however, our analysis revealed only limited spatial genetic structuring of its populations across the investigated area.

The results of the PCoA (Fig. 3) split the haplotypes of *O. filiformis* into four distinct clusters with notable separation. Among the 76 unique haplotypes identified, 19 were detected in multiple *Oswaldocruzia* specimens; however, there was no noticeable pattern regarding host association across these clusters, indicating a weak host-specific component in shaping the genetic variation of *O. filiformis*. Despite the lack of host-driven patterns, some geographic structure was observed, aligning with findings from previous studies [3, 7, 59, 84], further promoting the notion that nematodes exhibiting host generalist behavior are likely to display a weak population-genetic structure. Cluster A included almost all haplotypes from the Volga and Ural basins, but also encompassed haplotypes from the other two defined basins. Contrastingly, clusters B, C, and D were primarily composed of haplotypes from specimens collected within the Danube basin, suggesting a certain level of genetic differentiation within this vast geographical area. Additionally, three unique haplotypes from Greece were included in these clusters (the Ioannina Lake and Loutros River), suggesting potential interregional gene flow or historical connectivity between these locations and the Danube basin populations. Surprisingly, a unique haplotype (OF_28) was identified in the *O. filiformis* specimen from Ioannina Lake (Greece) which was distinctly separated from all four observed clusters (Supplementary Table S1). Significant divergence of the haplotype OF_28 may indicate the existence of a cryptic species in this region. As Gómez *et al.* [28] noted, cryptic species complexes often arise from recent, and sometimes rapid, speciation events. Such events might be ongoing in the Ioannina Lake, and it is possible that the new endemic haplotype (or cryptic species) emerged locally. All the other investigated specimens from this population carried OF_13, also present within cluster A. Further genetic and morphological analyses are needed to confirm whether the separation observed in OF_28 is associated with the existence of a distinct species or just reflects an intraspecific variation within *O. filiformis*.

Parasite distribution is directly influenced by the distribution of hosts [49]. However, to some extent, environmental factors may also contribute to the shaping of genetic differentiation among *O. filiformis* populations, although our results, concurrently with previous studies [44], suggest non-specific population-genetic patterns in this nematode species. Also, genetic diversity in amphibians, and potentially their parasites, is to some extent shaped by postglacial dispersal and lineage mixing as species recolonized deglaciated regions. The observed haplotype heterogeneity among the studied localities likely reflects these processes, highlighting the interconnected and continuous distribution of *O. filiformis* and its hosts from the Balkans. Its lifestyle, comprising both free-living and parasitic stages, offers multiple potential mechanisms for distribution. The nematode’s free-living stage enables independent movement (either on its own or long-range human-mediated [111]), while its parasitic stage relies on host species for dispersal. Furthermore, its wide host range, which includes amphibians, reptiles, and fish, enhances its dispersal potential across diverse ecological niches. The combination of a free-living life strategy, opportunistic parasitism, and a broad host range has likely allowed *O. filiformis* to utilize various dispersal routes and further facilitated its widespread distribution, and thus contributed to the observed genetic heterogeneity across the studied localities.

### Population genetic structure of *Oswaldocruzia filiformis* in Central Europe

Our comprehensive sampling campaign in Central Europe allowed us to study the population-genetic pattern on a small regional scale. Among the seven distribution groups defined by the major river basins in Slovakia, none of the haplotypes showed exclusive affiliation to a specific basin, instead appearing scattered randomly across all basins (Fig. 4). We would assume that at least in a region as geographically confined as Slovakia (and Czechia), there would be minimal genetic variation within the populations of *O. filiformis*. However, our results yet again revealed no associations between the obtained sequences and the potential distribution routes for amphibians associated with river basins. The observed distribution pattern of *O. filiformis* throughout Europe may be explained by the phylogeography of its host species. Previous studies have emphasized the importance of glacial and interglacial periods, as well as the subsequent recolonization of Central and northern Europe, in shaping species and genetic diversity in amphibians and reptiles (*e.g.*, [8, 40, 68, 110]). Southern Europe primarily served as a region of numerous microrefugia for amphibians and reptiles, promoting genetic diversification and the formation of unique and endemic genetic variants [21, 39]. While these major geoclimatic events shaped genetic variation in the hosts, the weak population-genetic structure observed in adult stages of *Oswaldocruzia* suggests the existence of additional drivers contributing to diversification in their nematode parasites.

Out of all 74 haplotypes identified in our dataset, *Oswaldocruzia* from Central Europe carried 46 haplotypes occurring only in this region (Table 2). However, this might be mainly due to sample bias, as a significantly larger number of frogs were examined from Central Europe compared to other regions, given that the frogs in Central Europe harbored more nematode individuals. This sample bias is also partially reflected in the distribution of haplotypes among host taxa, as the highest diversity was recorded among specimens collected from *B. bufo*. Most populations from Central Europe shared the haplotypes OF_1 and OF_6. The OF_1 haplotype was recorded not only from Central Europe but also from Albania, Bulgaria, and Greece. In contrast, the OF_6 haplotype was recorded only among populations in Central Europe. The widespread presence of haplotype OF_1 suggests historical or ongoing genetic flow between the mentioned regions, indicating a shared evolutionary history or dispersal events that connected these populations. In contrast, haplotype OF_6 represents localized genetic variation, likely shaped by regional ecological or environmental factors, which created this unique haplotype, probably thanks to limited dispersal or specific adaptation to the local environment. Further investigations of the ecological and evolutionary dynamics of *O. filiformis* and its interactions with host species are crucial for gaining deeper insights into the factors that have driven its distribution and genetic diversity. Additional genetic data on *O. filiformis* from a wider range of hosts and locations, together with sequence data on other species of the genus *Oswaldocruzia*, might shed more light on the diversification processes within this genus and elucidate the parasite’s shaping of host-parasite relationships in dynamic landscapes.

## Conclusion

*Oswaldocruzia* nematodes are common parasites of poikilothermic vertebrates. Although their species diversity is relatively well-documented, limited information is available regarding their host relationships at the genetic level and their phylogeography. These parasites exhibit varying levels of host specificity, ranging from strictly host-specific species, parasitizing a single host species, to true generalists such as O*. filiformis*. Our molecular data reveal that the specialist species *O. ukrainae*, which is closely associated with *B. viridis*, exhibits low genetic variability in the mitochondrial COI gene. In contrast, populations of *O. filiformis* in the western Palearctic exhibit an extremely high number of haplotypes. The distribution of these haplotypes indicates only weak population genetic structuring; however, their abundance highlights the elevated mutation rate in generalist nematode parasites. Notably, this high genetic variability is more pronounced at the nucleotide level compared to protein sequences, a fact that should be taken into account in future research.

## Supplementary material

The supplementary material for this article can be found at https://www.parasite-journal.org/10.1051/parasite/2025020/olm. *Supplementary Table S1*: A list of analyzed Oswaldocruzia spp. individuals with respective hosts and collection localities and COI GenBank accession numbers for each haplotype representative sequence. ID = specific code used for the respective *Oswaldocruzia* specimen; Haplotype = respective haplotype corresponding to Figures 2 and 4 and further discussed in the Results and Discussion sections; Cluster = position within multivariate space resulting from PCoA, as shown in Figure 3. Sequences retrieved from GenBank are marked by asterisks (*), sequences used for construction of phylogenetic tree are marked by a dagger (†). *Supplementary Table S2*: Individual host infections of Oswaldocruzia parasites at each collection site.
