# Supplementary material for: Insights into the genetic diversity and species distribution of Oswaldocruzia nematodes (Trichostrongylida: Molineidae) in Europe: apparent absence of geographic and population structuring in amphibians
Source: Parasite. 2025 Apr 23;32:27. doi: 10.1051/parasite/2025020 (PMC12021342; doi:10.1051/parasite/2025020)
Supplement: Supplementary file 1 — Supplementary Table S1: A list of analyzed Oswaldocruzia spp. individuals with respective hosts and collection localities and COI GenBank accession numbers for each haplotype representative sequence. ID = specific code used for the respective Oswaldocruzia specimen; Haplotype = respective haplotype corresponding to Figures 2 and 4 and further discussed in the Results and Discussion sections; Cluster = position within multivariate space resulting from PCoA, as shown in Figure 3. Sequences retrieved from GenBank are marked by asterisks (*), sequences used for construction of phylogenetic tree are marked by a dagger (†). [file parasite-32-27-s1.pdf]

Supplementary table S1. A list of analyzed *Oswaldocruzia* spp. individuals with respective hosts and collection localities and COI GenBank accession numbers for each haplotype representative sequence.

Oswaldocruzia species	Country	LocID	ID	Haplotype	Cluster	Host	Acc. Num. Cox1
Oswaldocruzia filiformis	Albania	DI	3204-A	OF_19	A	*Pelophylax shqipericus*	PV168589†
		QP	3219-A	OF_1	C	*Pelophylax kurtmuelleri*	PV168566
			3219-B	OF_60	A	*Pelophylax kurtmuelleri*	PV168607†
			3219-C	OF_5	A	*Pelophylax kurtmuelleri*	PV168570
	Bulgaria	KR	3736-A	OF_1	C	*Pelophylax* sp.	PV168566
			3736-B	OF_13	A	*Pelophylax* sp.	PV168585
			3736-C	OF_1	C	*Pelophylax* sp.	PV168566
	Czech Republic	BC	128/24-B	OF_3	C	*Bufo bufo*	PV168600
			128/24-C	OF_5	A	*Bufo bufo*	PV168570†
			129/24-A	OF_6	D	*Bufo bufo*	PV168616†
			129/24-B	OF_6	D	*Bufo bufo*	PV168616
	Greece	GR	4155-A	OF_1	C	*Pelophylax* sp.	PV168566
		CS	4359-A	OF_12	A	*Pelophylax* sp.	PV168584†
			4359-B	OF_13	A	*Pelophylax* sp.	PV168585
			4360-A	OF_14	A	*Pelophylax* sp.	PV168571†
		IO	3607-A	OF_13	A	*Pelophylax kurtmuelleri*	PV168585
			3607-B	OF_28	-	*Pelophylax kurtmuelleri*	PV168631†
			3608-A	OF_13	A	*Pelophylax kurtmuelleri*	PV168585
			3608-B	OF_13	A	*Pelophylax kurtmuelleri*	PV168585
			3611-A	OF_13	A	*Pelophylax epeiroticus*	PV168585
		LI	4447-A	OF_12	A	*Pelophylax* sp.	PV168584
			4447-B	OF_12	A	*Pelophylax* sp.	PV168584
			4449-A	OF_38	A	*Pelophylax* sp.	PV168602†
		LR	4138-A	OF_40	C	*Pelophylax* sp.	PV168594†
			4141-A	OF_9	B	*Pelophylax* sp.	PV168632
			4141-B	OF_1	C	*Pelophylax* sp.	PV168566
		MA	4184-A	OF_1	C	*Pelophylax* sp.	PV168566
			4184-B	OF_1	C	*Pelophylax* sp.	PV168566
			4191-A	OF_1	C	*Pelophylax* sp.	PV168566
			4191-B	OF_1	C	*Pelophylax* sp.	PV168566
			4192-A	OF_1	C	*Pelophylax* sp.	PV168566
		ZL	3633-A	OF_75	A	*Pelophylax kurtmuelleri*	PV168587†
			3633-B	OF_76	A	*Pelophylax kurtmuelleri*	PV168588†
	Romania	BM	4683-A	OF_10	C	*Pelophylax ridibundus*	PV168574†
			4688-A	OF_11	C	*Pelophylax ridibundus*	PV168575†
			4688-B	OF_9	B	*Pelophylax ridibundus*	PV168632†
		DU	4651-B	OF_10	C	*Pelophylax ridibundus*	PV168574
			4654-D	OF_21	A	*Pelophylax ridibundus*	PV168572†
			4654-E	OF_22	A	*Pelophylax ridibundus*	PV168586†
			4654-C	OF_13	A	*Pelophylax ridibundus*	PV168585
			4651-A	OF_10	C	*Pelophylax ridibundus*	PV168574
		ES	4586-A	OF_23	B	*Pelophylax ridibundus*	PV168630†
			4604-A	OF_24	B	*Pelophylax ridibundus*	PV168620†
			4604-B	OF_13	A	*Pelophylax ridibundus*	PV168585
		CV	4465-A	OF_15	A	*Pelophylax ridibundus*	PV168591†
	Slovakia	BR	B10-A	OF_1	C	*Bufo bufo*	PV168566†
			B10-B	OF_2	C	*Bufo bufo*	PV168599†
			B14-A	OF_3	C	*Bufo bufo*	PV168600†
			B14-B	OF_4	B	*Bufo bufo*	PV168608†
		BD	47/24-A	OF_4	B	*Bufo bufo*	PV168608
			47/24-B	OF_6	D	*Bufo bufo*	PV168616
			47/24-C	OF_7	D	*Bufo bufo*	PV168618†
			55/24-A	OF_8	A	*Bufo bufo*	PV168590†
		BU	18/23-A	OF_7	D	*Rana sp.*	PV168618
			18/23-B	OF_4	B	*Rana sp.*	PV168608
			19/23-B	OF_6	D	*Rana dalmatina*	PV168616
		CR	CPEES2-A	OF_16	C	*Pelophylax esculentus*	PV168601†
			CPEES2-B	OF_17	B	*Pelophylax esculentus*	PV168610†
			CPEES2-C	OF_4	B	*Pelophylax esculentus*	PV168608
			CPEES3-A	OF_6	D	*Pelophylax esculentus*	PV168616
		DV	DEVPERi1	OF_6	D	*Pelophylax ridibundus*	PV168616
			DEVPEES1	OF_4	B	*Pelophylax ridibundus*	PV168608
			DEVPERi5	OF_18	D	*Pelophylax ridibundus*	PV168624†
			DEVPERi10	OF_10	C	*Pelophylax ridibundus*	PV168574
		DH	13/23-B	OF_13	A	*Rana temporaria*	PV168585
			13/23-D	OF_6	D	*Rana temporaria*	PV168616
			15/23-B	OF_20	B	*Rana temporaria*	PV168619†
			13/23-C	OF_13	A	*Rana temporaria*	PV168585
		HS	02/23-A	OF_1	C	*Bufo bufo*	PV168566
			02/23-B	OF_3	C	*Bufo bufo*	PV168600
			02/23-C	OF_25	A	*Bufo bufo*	PV168568†
		HR	5/23-A	OF_13	A	*Bufo bufo*	PV168585
			5/23-B	OF_26	A	*Bufo bufo*	PV168573†
			4/23-B	OF_1	C	*Bufo bufo*	PV168566

HD	66/24-C	OF_27	A	*Rana temporaria*	PV168606†
	66/24-F	OF_6	D	*Rana temporaria*	PV168616
IJ	IJPeRi1-A	OF_31	D	*Pelophylax ridibundus*	PV168626†
	IJPERi3	OF_29	D	*Pelophylax ridibundus*	PV168617†
	IJPERi9	OF_30	B	*Pelophylax ridibundus*	PV168611†
JD	20/23-A	OF_7	D	*Rana dalmatina*	PV168618
	20/23-B	OF_7	D	*Rana dalmatina*	PV168618
	20/23-D	OF_32	A	*Rana dalmatina*	PV168595†
	20/23-E	OF_13	A	*Rana dalmatina*	PV168585
	20/23-F	OF_33	D	*Rana dalmatina*	PV168628†
KL	43/24-A	OF_7	D	*Bufo bufo*	PV168618
	43/24-B	OF_3	C	*Bufo bufo*	PV168600
	44/24-A	OF_6	D	*Bufo bufo*	PV168616
	44/24-B	OF_34	B	*Bufo bufo*	PV168612†
KVP	2/21-A	OF_37	B	*Bufotes viridis*	PV168613†
KV	46/24-A	OF_5	A	*Bufo bufo*	PV168570
	46/24-B	OF_6	D	*Bufo bufo*	PV168616
	56/24-B	OF_35	A	*Bufo bufo*	PV168605†
	61/24-A	OF_7	D	*Bufo bufo*	PV168618
KRL	KLPeRi2	OF_36	D	*Pelophylax ridibundus*	PV168621†
	KLPeRi4	OF_13	A	*Pelophylax ridibundus*	PV168585
KS	KS2-A	OF_3	C	*Bufo bufo*	PV168600
	KS2-B	OF_10	C	*Bufo bufo*	PV168574
LO	124/23-A	OF_1	C	*Bufo bufo*	PV168566
	124/23-B	OF_26	A	*Bufo bufo*	PV168573
	127/23-A	OF_1	C	*Bufo bufo*	PV168566
	132/23-A	OF_39	C	*Bufo bufo*	PV168576†
	132/23-B	OF_10	C	*Bufo bufo*	PV168574
ML	25/23-B	OF_16	C	*Bufo bufo*	PV168601
	28/23-A	OF_3	C	*Bufo bufo*	PV168600
	10/24-A	OF_7	D	*Rana dalmatina*	PV168618
	10/24-B	OF_41	C	*Rana dalmatina*	PV168581†
MV	6/24-A	OF_1	C	*Bufo bufo*	PV168566
	7/24-A	OF_42	C	*Bufo bufo*	PV168567†
	7/24-B	OF_10	C	*Bufo bufo*	PV168574
MD	MDRPeRi2-B	OF_10	C	*Pelophylax ridibundus*	PV168574
	MDRPeRi5-A	OF_43	C	*Pelophylax ridibundus*	PV168577†
	MDRPeRi5-B	OF_6	D	*Pelophylax ridibundus*	PV168616
MH	38/24-A	OF_10	C	*Bufo bufo*	PV168574
	38/24-B	OF_44	C	*Bufo bufo*	PV168598†
	40/24-A	OF_37	B	*Bufo bufo*	PV168613
MO	104/23-A	OF_45	C	*Bufo bufo*	PV168582†
	104/23-B	OF_1	C	*Bufo bufo*	PV168566
	62/24-A	OF_25	A	*Bufo bufo*	PV168568
MU	31/24-A	OF_25	A	*Bufo bufo*	PV168568
	32/24-A	OF_55	C	*Bufo bufo*	PV168603†
	32/24-B	OF_56	C	*Bufo bufo*	PV168583†
PCH	PCH2-A	OF_13	A	*Bufo bufo*	PV168585
	PCH13-A	OF_58	B	*Rana dalmatina*	PV168614†
	PCH13-B	OF_57	B	*Rana dalmatina*	PV168609†
PH	145/24-D	OF_7	D	*Bufotes viridis*	PV168618
PZ	3/23-A	OF_10	C	*Bufo bufo*	PV168574
	3/23-B	OF_6	D	*Bufo bufo*	PV168616
PS	136/24-A	OF_59	C	*Bufotes viridis*	PV168593†
RS	RUSPeES1	OF_6	D	*Pelophylax esculentus*	PV168616
	RUSPeES7	OF_1	C	*Pelophylax esculentus*	PV168566
RU	38/23-A	OF_61	C	*Bufo bufo*	PV168597†
	38/23-B	OF_3	C	*Bufo bufo*	PV168600
	45/23-A	OF_13	A	*Bufo bufo*	PV168585†
	47/23-A	OF_62	B	*Bufo bufo*	PV168622†
	47/23-B	OF_1	C	*Bufo bufo*	PV168566
SI	S19-A	OF_10	C	*Bufo bufo*	PV168574
	S19-C	OF_65	C	*Bufo bufo*	PV168569†
	S20-A	OF_66	C	*Bufo bufo*	PV168604†
	S20-B	OF_1	C	*Bufo bufo*	PV168566
	S45-A	OF_3	C	*Rana temporaria*	PV168600
	S45-B	OF_1	C	*Rana temporaria*	PV168566
	S45-C	OF_5	A	*Rana temporaria*	PV168570
	S46-A	OF_3	C	*Rana temporaria*	PV168600
	S46-B	OF_3	C	*Rana temporaria*	PV168600
SH	23/23-A	OF_1	C	*Rana temporaria*	PV168566
	23/23-B	OF_67	A	*Rana temporaria*	PV168596†
SS	SSPeES1-B	OF_63	C	*Pelophylax esculentus*	PV168578†
	SSPeES2	OF_34	B	*Pelophylax esculentus*	PV168612
	SSPeES3	OF_13	A	*Pelophylax esculentus*	PV168585
	SSPeES7	OF_64	B	*Pelophylax esculentus*	PV168629†
SR	ŠR1-A	OF_1	C	*Bufo bufo*	PV168566
	ŠR1-B	OF_3	C	*Bufo bufo*	PV168600
TS	26/24-B	OF_1	C	*Bufo bufo*	PV168566
	30/24-A	OF_1	C	*Bufo bufo*	PV168566
	30/24-B	OF_68	A	*Bufo bufo*	PV168592†

		VI	60/23-A	OF_13	A	*Bufo bufo*	PV168585
			70/23-A	OF_69	C	*Bufo bufo*	PV168579†
			87/23-A	OF_70	C	*Bufo bufo*	PV168580†
			73/24-A	OF_6	D	*Bufo bufo*	PV168616
		DN	49/24-A	OF_10	C	*Bufo bufo*	PV168574
		ZA	106/23-A	OF_6	D	*Bufo bufo*	PV168616
			109/23-A	OF_71	B	*Bufo bufo*	PV168627†
			111/23-A	OF_1	C	*Bufo bufo*	PV168566
		ZS	126/24-B	OF_62	B	*Bufo bufo*	PV168622
			119/24-A	OF_72	B	*Bufo bufo*	PV168623†
			123/24-A	OF_16	C	*Bufo bufo*	PV168601
			123/24-B	OF_73	B	*Bufo bufo*	PV168615†
			126/24-A	OF_74	D	*Bufo bufo*	PV168625†
	Russia	Mordovia Nature Reserve	Nem-Os-16	OF_47	A	*Lacerta agilis*	MT300266*
			Nem-Os-18	OF_48	A	*Lacerta agilis*	MT300262*†
			Nem-Os-10	OF_13	A	*Zootoca vivipara*	MT300260*
			Nem-Os-16_1	OF_53	A	*Zootoca vivipara*	MT300273*†
		Spassk city	Nem-Os-328-594	OF_15	A	*Bufotes viridis*	OQ346357*
		Samarskaya Luka	Nem-Os-116	OF_52	A	*Pelophylax ridibundus*	MT300271*†
			Nem-Os-120	OF_50	A	*Pelophylax ridibundus*	MT300269*†
			Nem-Os-183	OF_8	A	*Pelophylax ridibundus*	MT300272*
		Smolny National Park	Nem-Os-155	OF_47	A	*Bufo bufo*	MT300259*†
			Nem-Os-156	OF_5	A	*Rana arvalis*	MT300268*
			Nem-Os-88_4	OF_47	A	*Zootoca vivipara*	MT300267*
			Nem-Os-89_4	OF_8	A	*Lacerta agilis*	MT300275*
			Nem-Os-113	OF_49	A	*Vipera berus*	MT300263*†
			Nem-Os-123_4	OF_54	A	*Vipera berus*	MT300274*†
			Nem-Os-190_1	OF_47	A	*Anguis fragilis*	MT300264*
			Nem-Os-76_1	OF_47	A	*Natrix natrix*	MT300261*
			Nem-Os-107_17	OF_47	A	*Lacerta agilis*	MT300265*
			Nem-Os-95_5	OF_51	A	*Lacerta agilis*	MT300270*†
		Uzola floodplain	Nem-Os-157	OF_1	C	*Rana arvalis*	MT300257*
		Ural floodplain	Nem-Os-158	OF_46	A	*Rana arvalis*	MT300256*†
		Zvenigorod Biological Station of Moscow University	Nem-Os-159	OF_10	C	*Rana temporaria*	MT300258*
Oswaldocruzia ukrainae	Slovakia	KVP	104/24-A	-	-	*Bufotes viridis*	PV168633†
		MP	24/23-B	-	-	*Bufotes viridis*	PV168633†
			89/24-A	-	-	*Bufotes viridis*	PV168633†
			24/23-C	-	-	*Bufotes viridis*	PV168633†
		PH	145/24-A	-	-	*Bufotes viridis*	PV168633†
			145/24-C	-	-	*Bufotes viridis*	PV168633†
	Russia	Samara city	Nem-Os-329-595	-	-	*Bufotes viridis*	OQ351734*†
			Nem-Os-335-601	-	-	*Bufotes viridis*	OQ351739*†
		Lykovshchina village	Nem-Os-331-597	-	-	*Bufotes viridis*	OQ351735*†
			Nem-Os-332-598	-	-	*Bufotes viridis*	OQ351736*†
		Nikolaevka village	Nem-Os333-599	-	-	*Bufotes viridis*	OQ351737*†
			Nem-Os-334-600	-	-	*Bufotes viridis*	OQ351738*†
Oswalocruzia sp.	Italy	Cocullo	CS071	-	-	*Elaphe quatuorlineata*	OR761977*†
Ancylostoma tubaeforme	China	Guangzhou	-	-	-	Stray cat	NC_034289†
Ancylostoma ceylanicum	Vietnam	Long An Province	-	-	-	Humans	MW549613†

ID = specific code used for the respective *Oswaldocruzia* specimen; Haplotype = respective haplotype corresponding to Figures 2 and 4 and further discussed in the Results and Discussion section; Cluster = position within multivariate space resulting from PCoA as shown in Figure 3. Sequences retrieved from GenBank are marked by asterisks (*), sequences used for construction of phylogenetic tree are marked by cross (†).
