# Supplementary material for: Insights into the genetic diversity and species distribution of Oswaldocruzia nematodes (Trichostrongylida: Molineidae) in Europe: apparent absence of geographic and population structuring in amphibians
Source: Parasite. 2025 Apr 23;32:27. doi: 10.1051/parasite/2025020 (PMC12021342; doi:10.1051/parasite/2025020)
Supplement: Supplementary file 2 — Supplementary Table S2: Individual host infections of Oswaldocruzia parasites at each collection site. [file parasite-32-27-s2.pdf]

Supplementary table S2. The individual host infections of *Oswaldocruzia* parasites on each collection sites.

Country	Locality	LocID	Y	X	Speciment_ID	Host_species	Host species sex	N Oswald.
Albania	Divjake	DI	40.990129	19.497491	3161	*P. kurtmuelleri*		
					3165	*P. kurtmuelleri*	Female	
					3169	*P. kurtmuelleri*	Male	
					3170	*P. kurtmuelleri*	Male	
					3172	*P. kurtmuelleri*	Female	
					3173	*P. kurtmuelleri*	Female	
					3174	*P. kurtmuelleri*	Male	
					3175	*P. kurtmuelleri*	Female	
					3176	*P. kurtmuelleri*	Male	
					3177	*P. kurtmuelleri*	Female	
					3196	*P. shqipericus*	Male	
					3202	*P. shqipericus*	Male	
					3203	*P. shqipericus*	Female	
					3204	*P. shqipericus*	Male	1
	Qazim Pali	QP	40.049669	19.841988	3208	*P. kurtmuelleri*	Female	
					3213	*P. kurtmuelleri*	Male	
					3215	*P. kurtmuelleri*	Male	
					3216	*P. kurtmuelleri*	Male	
					3217	*P. kurtmuelleri*	Male	
					3218	*P. kurtmuelleri*	Male	
					3219	*P. kurtmuelleri*	Male	3
					3220	*P. kurtmuelleri*	Female	
					3231	*P. kurtmuelleri*	Male	
	Xarrë	XA	39.733001	20.052892	3240	*P. kurtmuelleri*	Male	
Bulgaria	Kremenik	KR	42.360640	23.074380	3732	*Pelophylax* sp.	Male	
					3735	*Pelophylax* sp.	Male	
					3736	*Pelophylax* sp.	Male	10
					3738	*Pelophylax* sp.	Male	
					3740	*Pelophylax* sp.	Male	
Czech Republic	Buchlovice	BC	49.086642	17.337341	127/24	*B. bufo*	Female	
					128/24	*B. bufo*	Male	23
					129/24	*B. bufo*	Male	15
Greece	Gravouna	GR	41.000261	24.670300	4148	*Pelophylax* sp.	Female	
					4149	*Pelophylax* sp.	Male	
					4150	*Pelophylax* sp.	Male	
					4151	*Pelophylax* sp.	Male	
					4152	*Pelophylax* sp.	Male	
					4153	*Pelophylax* sp.	Male	
					4154	*Pelophylax* sp.	Male	
					4155	*Pelophylax* sp.	Female	1
					4156	*Pelophylax* sp.	Male	
					4157	*Pelophylax* sp.	Male	
	Cheimaros	CS	41.120780	23.252460	4359	*Pelophylax* sp.	Male	2
					4360	*Pelophylax* sp.	Female	1
					4361	*Pelophylax* sp.	Female	
					4363	*Pelophylax* sp.	Female	
					4365	*Pelophylax* sp.	Male	
	Igoumenitsa	IG	39.536593	20.203803	3248	*P. epeiroticus*	Female	
					3254	*P. epeiroticus*	Male	
					3259	*P. epeiroticus*	Male	
					3263	*P. epeiroticus*	Male	
					3264	*P. epeiroticus*	Male	
					3265	*P. kurtmuelleri*	Female	2
					3271	*P. epeiroticus*	Male	
					3273	*P. eperoticus*	Female	
					3275	*P. epeiroticus*	Male	
					3278	*P. kurtmuelleri*	Female	
					3281	*P. epeiroticus*	Male	
					3282	*P. kurtmuelleri*	Female	
					3283	*P. epeiroticus*	Male	
	Ioannina	IO	39.688624	20.858381	3284	*P. epeiroticus*	Female	
					3291	*P. epeiroticus*	Female	
					3299	*P. epeiroticus*	Female	
					3302	*P. epeiroticus*	Female	
					3303	*P. epeiroticus*	Female	
					3310	*P. epeiroticus*	Female	
					3607	*P. kurtmuelleri*	Male	24
					3608	*P. kurtmuelleri*	Female	7
					3614	*P. epeiroticus*	Female	
					3611	*P. epeiroticus*	Male	1
					3615	*P. epeiroticus*	Female	
					3609	*P. epeiroticus*	Male	
	Limanaki	LI	38.172760	21.421850	3652	*P. epeiroticus*	Male	2
					3653	*P. epeiroticus*	Female	
					3654	*P. epeiroticus*	Male	
					3655	*P. epeiroticus*	Male	
					3657	*P. epeiroticus*	Male	
					3656	*P. epeiroticus*	Male	
					3658	*P. epeiroticus*	Male	

					3673	*P. epeiroticus*	Male	
					3674	*P. epeiroticus*	Male	
					3675	*P. epeiroticus*	Male	
	Lithotopos	LT	41.138280	23.219900	4440	*Pelophylax* sp.	Female	
					4445	*Pelophylax* sp.	Female	
					4446	*Pelophylax* sp.	Female	1
					4447	*Pelophylax* sp.	Male	5
					4448	*Pelophylax* sp.	Female	
					4449	*Pelophylax* sp.	Female	6
					4450	*Pelophylax* sp.	Male	4
					4451	*Pelophylax* sp.	Male	1
					4452	*Pelophylax* sp.	Male	
					4453	*Pelophylax* sp.	Female	
	Loutros River	LR	40.865230	26.032050	4138	*Pelophylax* sp.	Male	3
					4139	*Pelophylax* sp.	Female	
					4140	*Pelophylax* sp.	Female	1
					4141	*Pelophylax* sp.	Male	4
					4142	*Pelophylax* sp.	Male	2
					4143	*Pelophylax* sp.	Female	
					4144	*Pelophylax* sp.	Female	
					4145	*Pelophylax* sp.	Male	3
					4146	*Pelophylax* sp.	Female	2
					4147	*Pelophylax* sp.	Male	4
	Mavrolefki	MA	41.047350	24.094550	4177	*Pelophylax* sp.	Male	
					4178	*Pelophylax* sp.	Male	
					4179	*Pelophylax* sp.	Female	
					4180	*Pelophylax* sp.	Female	
					4181	*Pelophylax* sp.	Male	
					4182	*Pelophylax* sp.	Female	2
					4184	*Pelophylax* sp.	Male	4
					4185	*Pelophylax* sp.	Male	
					4186	*Pelophylax* sp.	Male	1
					4187	*Pelophylax* sp.	Male	
					4188	*Pelophylax* sp.	Male	
					4189	*Pelophylax* sp.	Male	
					4190	*Pelophylax* sp.	Male	
					4191	*Pelophylax* sp.	Male	6
					4192	*Pelophylax* sp.	Female	3
					4193	*Pelophylax* sp.	Male	
					4194	*Pelophylax* sp.	Male	
					4195	*Pelophylax* sp.	Female	
					4196	*Pelophylax* sp.	Male	1
	Prosotsani	PR	41.169279	23.972630	4209	*Pelophylax* sp.	Female	
					4210	*Pelophylax* sp.	Female	
					4211	*Pelophylax* sp.	Female	
					4212	*Pelophylax* sp.	Female	
					4213	*Pelophylax* sp.	Male	
					4214	*Pelophylax* sp.	Male	
					4215	*Pelophylax* sp.	Male	
					4216	*Pelophylax* sp.	Female	
					4217	*Pelophylax* sp.	Female	
					4218	*Pelophylax* sp.	Female	
	Zirou lake	ZL	39.240600	20.853900	3617	*P. kurtmuelleri*	Female	
					3618	*P. epeiroticus*	Male	
					3619	*P. kurtmuelleri*	Female	
					3627	*P. kurtmuelleri*	Male	
					3633	*P. kurtmuelleri*	Female	2
					3620	*P. epeiroticus*	Male	
					3628	*P. kurtmuelleri*	Male	
					3622	*P. kurtmuelleri*	Female	3
					3623	*P. kurtmuelleri*	Male	1
					3626	*P. kurtmuelleri*	Male	
					3624	*P. kurtmuelleri*	Male	
					3625	*P. kurtmuelleri*	Male	
Romania	Budeasa Mică	BM	44.903817	24.846980	4679	*P. ridibundus*	Female	
					4680	*P. ridibundus*	Female	
					4681	*P. ridibundus*	Male	
					4682	*P. ridibundus*	Male	
					4683	*P. ridibundus*	Male	1
					4684	*P. ridibundus*	Male	
					4685	*P. ridibundus*	Female	
					4686	*P. ridibundus*	Male	
					4687	*P. ridibundus*	Male	
					4688	*P. ridibundus*	Male	2
					4689	*P. ridibundus*	Male	
	Dumbrăviţa	DU	45.767861	25.458950	4648	*P. ridibundus*	Male	
					4649	*P. ridibundus*	Female	
					4651	*P. ridibundus*	Male	2
					4652	*P. ridibundus*	Female	
					4653	*P. ridibundus*	Male	1
					4654	*P. ridibundus*	Male	6

					4655	*P. ridibundus*	Male	
					4656	*P. ridibundus*	Female	1
					4657	*P. ridibundus*	Female	
					4658	*P. ridibundus*	Male	
	Eşelniţa	ES	44.686268	22.368010	4586	*P. ridibundus*	Female	3
					4587	*P. ridibundus*	Male	3
					4588	*P. ridibundus*	Male	
					4595	*P. ridibundus*	Male	
					4599	*P. ridibundus*	Male	
					4600	*P. ridibundus*	Male	1
					4601	*P. ridibundus*	Male	
					4602	*P. ridibundus*	Male	14
					4603	*P. ridibundus*	Male	1
					4604	*P. ridibundus*	Male	7
	Chilia Veche	CV	45.418647	29.276885	4456	*P. ridibundus*	Female	
					4457	*P. ridibundus*	Male	
					4458	*P. ridibundus*	Female	
					4459	*P. ridibundus*	Male	
					4460	*P. ridibundus*	Female	
					4461	*P. ridibundus*	Male	1
					4462	*P. ridibundus*	Female	
					4463	*P. ridibundus*	Female	1
					4464	*P. ridibundus*	Male	
					4465	*P. ridibundus*	Female	1
					4466	*P. ridibundus*	Female	
Slovakia	Brestov	BR	48.869871	21.348455	B1	*B. bufo*		9
					B2	*B. bufo*		20
					B3	*B. bufo*		27
					B4	*B. bufo*		8
					B5	*B. bufo*		4
					B6	*B. bufo*		5
					B7	*B. bufo*		25
					B8	*B. bufo*		5
					B9	*B. bufo*		12
					B10	*B. bufo*		12
					B11	*B. bufo*		6
					B12	*B. bufo*		20
					B13	*B. bufo*		9
					B14	*B. bufo*		6
					B15	*B. bufo*		4
	Budča	BD	48.574399	19.069150	47/24	*B. bufo*	Male	3
					48/24	*B. bufo*	Male	
					55/24	*B. bufo*	Male	1
	Budiš	BU	48.874002	18.748319	16/23	*R. dalmatina*		
					17/23	*R. dalmatina*		
					18/23	*Rana* sp.		14
					19/23	*R. dalmatina*		4
					21/23	*R. dalmatina*		3
	Cestice	CE	48.590383	21.100551	110/24	*B. viridis*	Male	
					111/24	*B. viridis*	Male	
					112/24	*B. viridis*	Female	
					113/24	*B. viridis*		
					114/24	*B. viridis*		
	Čečejovce	CC	48.598886	21.062267	90/24	*B. viridis*	Female	
					91/24	*B. viridis*	Female	
					92/24	*B. viridis*	Female	
					93/24	*B. viridis*	Male	
					94/24	*B. viridis*	Female	1
	Číčovský stream	CR	47.764833	17.728222	CPEES1	*P. esculentus*	Male	
					CPEES2	*P. esculentus*		8
					CPEES3	*P. esculentus*		1
					CPEES4	*P. esculentus*		
					CPEES5	*P. esculentus*		
					CPEES6	*P. esculentus*		
					CPEES7	*P. esculentus*		
	Demjata	DE	49.107776	21.311242	147/24	*B. viridis*	Female	
	Devín	DV	48.174944	16.976694	DEVPeRi1	*P. ridibundus*	Male	
					DEVPeRi2	*P. ridibundus*	Male	3
					DEVPeRi3	*P. ridibundus*	Female	3
					DEVPeRi4	*P. ridibundus*	Male	
					DEVPeRi5	*P. ridibundus*	Female	4
					DEVPeRi6	*P. ridibundus*	Male	1
					DEVPeRi7	*P. ridibundus*	Male	
					DEVPeRi8	*P. ridibundus*	Male	
					DEVPeRi9	*P. ridibundus*	Male	
					DEVPeRi10	*P. ridibundus*	Female	3
					DEVPeRi11	*P. ridibundus*	Male	2
					DEVPeEs1	*P. esculentus*	Male	2
					DEVPeEs2	*P. esculentus*	Female	
					DEVPeEs3	*P. esculentus*	Male	
					DEVPeEs4	*P. esculentus*	Female	

				DEVPeEs5	*P. esculentus*	Female	1
				DEVPeEs6	*P. esculentus*	Female	
				DEVPeEs7	*P. esculentus*	Female	
				DEVPeEs8	*P. esculentus*	Female	1
				DEVPeEs9	*P. esculentus*	Female	
				DEVPeEs10	*P. esculentus*	Male	
Dobrá Niva	DN	48.471648	19.102409	49/24	*B. bufo*	Male	1
				57/24	*B. bufo*	Male	
Dolný Harmanec	DH	48.814759	19.049393	7/23	*R. dalmatina*		
				8/23	*R. temporaria*		
				9/23	*R. dalmatina*		
				10/23	*R. temporaria*		
				11/23	*R. temporaria*		1
				12/23	*R. dalmatina*		1
				13/23	*R. temporaria*		5
				14/23	*R. temporaria*		
				15/23	*R. temporaria*		3
Gbelce	GB	47.858444	18.508861	GPeRi1	*P. ridibundus*		
				GPeRi2	*P. ridibundus*		
				GPeRi3	*P. ridibundus*		
				GPeRi4	*P. ridibundus*		
				GPeRi5	*P. ridibundus*		
				GPeRi6	*P. ridibundus*		
				GPeRi7	*P. ridibundus*		
				GPeRi8	*P. ridibundus*		
				GPeRi9	*P. ridibundus*		
				GPeRi10	*P. ridibundus*		
				GPeRi11	*P. ridibundus*		
				GPeRi15	*P. ridibundus*	Male	
Horná Štubňa	HS	48.823706	18.901571	2/23	*B. bufo*		9
Hrabovo	HR	49.072325	19.274079	4/23	*B. bufo*		2
				5/23	*B. bufo*		17
				6/23	*B. bufo*		
Hronská Dubrava	HD	48.589677	19.001009	66/24	*R. temporaria*	Female	10
Chmeľovec	CH	49.082935	21.371885	146/24	*B. viridis*	Male	
Chorvátsky stream	CHR	48.099861	17.129694	CHPeRi1	*P. ridibundus*		
				CHPeRi2	*P. ridibundus*		
				CHPeRi3	*P. ridibundus*		
				CHPeRi4	*P. ridibundus*		
				CHPeRi5	*P. ridibundus*		
				CHPeRi6	*P. ridibundus*		
				CHPeRi7	*P. ridibundus*		
				CHPeRi8	*P. ridibundus*		
				CHPeRi9	*P. ridibundus*		
				CHPeRi10	*P. ridibundus*		
Ivánka pri Dunaji - Lake	IJ	48.175972	17.262556	IJPeRi1	*P. ridibundus*	Male	11
				IJPeRi2	*P. ridibundus*	Female	
				IJPeRi3	*P. ridibundus*	Female	
				IJPeRi4	*P. ridibundus*	Male	
				IJPeRi5	*P. ridibundus*	Male	
				IJPeRi6	*P. ridibundus*	Female	1
				IJPeRi7	*P. ridibundus*	Female	
				IJPeRi8	*P. ridibundus*	Female	
				IJPeRi9	*P. ridibundus*	Male	1
				IJPeRi10	*P. ridibundus*	Male	1
Jasenská Dolina	JD	48.861102	19.455961	20/23	*R. dalmatina*		23
Kolačno	KL	48.591803	18.421305	41/24	*B. bufo*	Female	
				42/24	*B. bufo*	Female	5
				43/24	*B. bufo*	Female	19
				44/24	*B. bufo*	Female	18
				45/24	*B. bufo*	Male	
				67/24	*B. bufo*	Female	2
				68/24	*B. bufo*	Female	17
				69/24	*B. bufo*	Female	
Košice - KVP	KVP	48.715139	21.211511	1/21	*B. viridis*		1
				2/21	*B. viridis*		4
				3/21	*B. viridis*		3
				4/21	*B. viridis*		
				5/21	*B. viridis*		
				100/24	*B. viridis*	Female	
				101/24	*B. viridis*	Male	
				102/24	*B. viridis*	Male	
				103/24	*B. viridis*	Male	
				104/24	*B. viridis*	Male	1
Košice - City Park	MP	48.723888	21.265415	24/23	*B. viridis*		30
				85/24	*B. viridis*	Female	1
				86/24	*B. viridis*	Male	4
				87/24	*B. viridis*	Male	
				88/24	*B. viridis*	Male	
				89/24	*B. viridis*	Male	1
Košice - Zuzka's park	ZP	48.718835	21.238002	105/24	*B. viridis*	Male	

				106/24	*B. viridis*	Male	
				107/24	*B. viridis*	Male	
				108/24	*B. viridis*	Male	
				109/24	*B. viridis*	Male	
Kováčová	KV	48.601779	19.104349	46/24	*B. bufo*	Male	8
				52/24	*B. bufo*	Male	1
				53/24	*B. bufo*	Male	1
				54/24	*B. bufo*	Female	
				56/24	*B. bufo*	Male	24
				61/24	*B. bufo*	Male	3
				64/24	*B. bufo*	Male	3
				65/24	*B. bufo*	Male	
Kráľovská lúka	KRL	47.898389	17.481639	KLPeRi1	*P. ridibundus*	Male	
				KLPeRi2	*P. ridibundus*		
				KLPeRi3	*P. ridibundus*		
				KLPeRi4	*P. ridibundus*		
				KLPeRi5	*P. ridibundus*		
				KLPeRi6	*P. ridibundus*		
				KLPeRi7	*P. ridibundus*		
				KLPeRi8	*P. ridibundus*		
				KLPeRi9	*P. ridibundus*		
				KLPeRi10	*P. ridibundus*		
				KLPeEs1	*P. ridibundus*		
				KLPeEs2	*P. ridibundus*		
				KLPeEs3	*P. ridibundus*		
Kusín	KS	48.813688	22.063373	KS1	*R. dalmatina*		1
				KS2	*B. bufo*		5
				KS3	*B. bufo*		17
Lipovec	LP	49.123771	18.933342	1/23	*B. bufo*		
Lomné	LO	49.106705	21.636617	118/23	*R. temporaria*		
				119/23	*R. temporaria*		
				120/23	*R. temporaria*		
				121/23	*R. temporaria*		
				122/23	*B. bufo*	Male	3
				123/23	*B. bufo*	Male	2
				124/23	*B. bufo*	Female	6
				125/23	*B. bufo*	Male	2
				126/23	*B. bufo*	Male	2
				127/23	*B. bufo*	Male	5
				128/23	*B. bufo*	Male	1
				129/23	*B. bufo*	Male	
				130/23	*B. bufo*	Male	7
				131/23	*B. bufo*	Male	1
				132/23	*B. bufo*	Female	9
				133/23	*B. bufo*	Male	
				134/23	*B. bufo*		
				135/23	*B. bufo*	Female	
				136/23	*B. bufo*	Male	
				137/23	*B. bufo*	Female	2
Malá Lodina	ML	48.875902	21.139193	25/23	*B. bufo*		15
				27/23	*B. bufo*		1
				28/23	*B. bufo*		11
				29/23	*B. bufo*		5
				30/23	*B. bufo*	Male	4
				31/23	*B. bufo*		3
				32/23	*B. bufo*	Male	6
				33/23	*B. bufo*		1
				34/23	*B. bufo*		4
				35/23	*B. bufo*	Female	1
				36/23	*B. bufo*	Female	17
				1/24	*R. temporaria*		
				2/24	*R. temporaria*		
				3/24	*R. temporaria*		
				4/24	*R. temporaria*		
				5/24	*B. bufo*	Male	
				10/24	*R. dalmatina*	Male	2
				11/24	*B. bufo*	Male	4
				12/24	*R. dalmatina*	Female	
				13/24	*R. dalmatina*	Female	
				14/24	*R. dalmatina*	Female	
				18/24	*B. bufo*	Female	
				19/24	*B. bufo*	Male	1
				121/24	*R. dalmatina*	Female	
Malá Vieska	MV	48.801711	21.246164	6/24	*B. bufo*	Male	5
				7/24	*B. bufo*	Male	7
				8/24	*B. bufo*	Male	8
				9/24	*B. bufo*	Male	8
Malý Draždiak	MD	48.108917	17.119361	MDRPeRi1	*P. ridibundus*	Male	
				MDRPeRi2	*P. ridibundus*	Female	1
				MDRPeRi3	*P. ridibundus*	Male	
				MDRPeRi4	*P. ridibundus*	Male	

				MDRPeRi5	*P. ridibundus*	Male	36
Modra-Harmonia	MH	48.360295	17.307702	38/24	*B. bufo*	Female	7
				39/24	*B. bufo*	Female	48
				40/24	*B. bufo*	Female	8
Mokrance	MK	48.593335	21.022273	95/24	*B. viridis*	Male	
				96/24	*B. viridis*	Male	
				97/24	*B. viridis*	Female	
				98/24	*B. viridis*	Male	
				99/24	*B. viridis*	Male	
Môťová	MO	48.553451	19.175150	99/23	*B. bufo*	Female	
				100/23	*B. bufo*	Male	5
				101/23	*B. bufo*	Male	
				102/23	*B. bufo*	Male	13
				103/23	*B. bufo*	Male	2
				104/23	*B. bufo*	Male	25
				50/24	*B. bufo*	Male	5
				51/24	*B. bufo*	Male	10
				58/24	*B. bufo*	Male	2
				59/24	*B. bufo*	Male	9
				60/24	*B. bufo*	Male	
				62/24	*B. bufo*	Female	8
				63/24	*B. bufo*	Male	
Muránska Lehota	MU	48.728261	20.048167	15/24	*B. bufo*	Male	
				16/24	*B. bufo*	Male	
				17/24	*B. bufo*	Male	1
				24/24	*B. bufo*	Male	
				31/24	*B. bufo*	Male	9
				32/24	*B. bufo*	Male	3
				33/24	*B. bufo*	Male	
Párnica	PA	49.199106	19.184862	91/23	*R. temporaria*	Female	
				92/23	*R. temporaria*	Female	3
				93/23	*R. temporaria*	Female	
				94/23	*R. temporaria*	Male	1
				95/23	*R. temporaria*	Male	
				96/23	*R. temporaria*		
				97/23	*R. temporaria*		
				98/23	*R. temporaria*	Female	
Perín-Chým	PCH	48.534557	21.187797	PCH1	*B. bufo*		3
				PCH2	*B. bufo*		6
				PCH3	*B. bufo*		5
				PCH4	*B. bufo*		3
				PCH5	*B. bufo*		7
				PCH6	*B. bufo*		5
				PCH7	*B. bufo*		4
				PCH8	*B. bufo*		
				PCH9	*B. bufo*		10
				PCH10	*B. bufo*		32
				PCH11	*B. bufo*		17
				PCH12	*B. bufo*		8
				PCH13	*B. bufo*		9
				PCH14	*B. bufo*		6
				PCH15	*B. bufo*		
				PCH16	*B. bufo*		1
				PCH17	*B. bufo*		4
				PCH18	*B. bufo*		1
				PCH19	*B. bufo*		3
				PCH20	*B. bufo*		
				PCH21	*B. bufo*		9
				PCH22	*B. bufo*		12
				PCH23	*B. bufo*		
				PCH24	*B. bufo*		
				PCH25	*B. bufo*		4
				PCH26	*B. bufo*		12
				PCH27	*B. bufo*		3
				PCH28	*B. bufo*		1
				PCH29	*R. dalmatina*		1
				PCH30	*R. dalmatina*		3
				PCH31	*R. dalmatina*		
				PCH32	*R. dalmatina*		2
				PCH33	*R. dalmatina*		
				PCH34	*R. dalmatina*		
				PCH35	*R. dalmatina*		
				PCH36	*R. dalmatina*		
Podhorany	PH	49.083212	21.356049	142/24	*B. viridis*	Female	
				143/24	*B. viridis*	Female	
				144/24	*B. viridis*	Female	
				145/24	*B. viridis*	Female	4
Požehy	PZ	48.844820	18.814701	3/23	*B. bufo*		2
Prešov-city square	PKP	49.006469	21.224027	137/24	*B. viridis*	Female	
				138/24	*B. viridis*	Female	
				139/24	*B. viridis*	Male	

				140/24	*B. viridis*	Male	
				141/24	*B. viridis*	Male	
Prešov-Šváby	PS	48.971488	21.266167	132/24	*B. viridis*	Male	
				133/24	*B. viridis*	Female	
				134/24	*B. viridis*	Female	
				135/24	*B. viridis*	Female	
				136/24	*B. viridis*	Male	1
Rad site Hrušov	RH	48.435857	21.861214	80/24	*B. viridis*	Male	
				81/24	*B. viridis*	Female	
				82/24	*B. viridis*	Male	2
				83/24	*B. viridis*	Female	
				84/24	*B. viridis*	Male	
Rudník Lake	SR	48.702849	21.008252	SR1	*B. bufo*		2
Rusovce	RS	48.057444	17.153111	RUSPeEs1	*P. esculentus*		
				RUSPeEs2	*P. esculentus*		
				RUSPeEs3	*P. esculentus*		
				RUSPeEs4	*P. esculentus*		
				RUSPeEs5	*P. esculentus*		
				RUSPeEs6	*P. esculentus*		
				RUSPeEs7	*P. esculentus*		6
				RUSPeEs8	*P. esculentus*		
				RUSPeEs9	*P. esculentus*		1
				RUSPeEs10	*P. esculentus*		1
Ružín	RU	48.822152	21.081126	26/23	*B. bufo*		25
				37/23	*B. bufo*	Female	1
				38/23	*B. bufo*	Female	36
				39/23	*B. bufo*	Female	8
				40/23	*B. bufo*	Male	4
				41/23	*B. bufo*	Male	6
				42/23	*B. bufo*	Male	12
				43/23	*B. bufo*	Male	2
				44/23	*B. bufo*	Male	4
				45/23	*B. bufo*	Male	18
				46/23	*B. bufo*	Male	1
				47/23	*B. bufo*	Female	21
				48/23	*B. bufo*	Male	
				49/23	*B. bufo*	Male	6
				50/23	*B. bufo*	Male	6
				51/23	*B. bufo*	Male	11
				52/23	*B. bufo*	Female	8
				53/23	*B. bufo*	Male	10
				54/23	*B. bufo*	Male	3
				55/23	*B. bufo*	Male	17
				56/23	*B. bufo*	Male	
				57/23	*B. bufo*	Male	
Sigord	SI	48.950347	21.353007	SI1	*B. bufo*		29
				SI2	*B. bufo*		3
				SI3	*B. bufo*		20
				SI4	*B. bufo*		19
				SI5	*B. bufo*		11
				SI6	*B. bufo*		8
				SI7	*B. bufo*		25
				SI8	*B. bufo*		49
				SI9	*B. bufo*		13
				SI10	*B. bufo*		12
				SI11	*B. bufo*		19
				SI12	*B. bufo*		23
				SI13	*B. bufo*		19
				SI14	*B. bufo*		24
				SI15	*B. bufo*		34
				SI16	*B. bufo*		23
				SI17	*B. bufo*		17
				SI18	*B. bufo*		16
				SI19	*B. bufo*		7
				SI20	*B. bufo*		25
				SI21	*B. bufo*		6
				SI22	*B. bufo*		1
				SI23	*B. bufo*		13
				SI24	*B. bufo*		11
				SI25	*B. bufo*		33
				SI26	*B. bufo*		17
				SI27	*B. bufo*		3
				SI28	*B. bufo*		2
				SI29	*B. bufo*		3
				SI30	*B. bufo*		4
				SI31	*B. bufo*		10
				SI32	*B. bufo*		7
				SI33	*B. bufo*		2
				SI34	*B. bufo*		4
				SI35	*B. bufo*		8
				SI36	*B. bufo*		8

				SI37	*B. bufo*		
				SI38	*B. bufo*		12
				SI39	*B. bufo*		17
				SI40	*B. bufo*		19
				SI41	*B. bufo*		14
				SI42	*B. bufo*		14
				SI43	*B. bufo*		11
				SI44	*R. temporaria*		9
				SI45	*R. temporaria*		
				SI46	*R. temporaria*		8
				SI47	*R. temporaria*		4
				SI48	*R. temporaria*		
				SI49	*R. temporaria*		
				SI50	*R. dalmatina*		
Staré Hory	SH	48.831426	19.102745	22/23	*Rana* sp.		
				23/23	*R. temporaria*		18
Šaštín-Stráže	SS	48.632139	17.139361	SSPeEs1	*P. esculentus*	Female	3
				SSPeEs2	*P. esculentus*	Female	1
				SSPeEs3	*P. esculentus*	Female	2
				SSPeEs4	*P. esculentus*	Female	
				SSPeEs5	*P. esculentus*	Female	6
				SSPeEs6	*P. esculentus*	Male	1
				SSPeEs7	*P. esculentus*	Female	9
				SSPeEs8	*P. esculentus*	Female	
				SSPeEs9	*P. esculentus*	Male	
				SSPeEs10	*P. esculentus*	Female	
Šulianske Lake	SJ	47.946167	17.423222	SULPeRi1	*P. ridibundus*	Female	
				SULPeRi2	*P. ridibundus*		
				SULPeRi3	*P. ridibundus*		
				SULPeRi4	*P. ridibundus*	Male	
				SULPeRi5	*P. ridibundus*	Male	
				SULPeRi6	*P. ridibundus*	Male	
				SULPeRi7	*P. ridibundus*	Male	
				SULPeRi8	*P. ridibundus*	Male	
				SULPeRi9	*P. ridibundus*	Female	
				SULPeRi10	*P. ridibundus*	Male	
Tisovec	TS	48.68096	19.943049	20/24	*B. bufo*	Female	4
				21/24	*B. bufo*	Female	
				22/24	*B. bufo*	Male	3
				23/24	*B. bufo*	Male	
				25/24	*B. bufo*	Male	16
				26/24	*B. bufo*	Female	20
				27/24	*B. bufo*	Male	6
				28/24	*B. bufo*	Male	1
				29/24	*B. bufo*	Male	2
				30/24	*B. bufo*	Male	20
				34/24	*B. bufo*	Female	1
				35/24	*B. bufo*	Female	
				36/24	*B. bufo*	Male	
				37/24	*B. bufo*	Female	7
Veľký Lél - Island	VLO	47.754861	17.944639	LPeEs1	*P. esculentus*		
				LPeEs2	*P. esculentus*		
				LPeEs3	*P. esculentus*		3
				LPeEs4	*P. esculentus*		
				LPeEs5	*P. esculentus*		
				LPeEs6	*P. esculentus*		
				LPeEs7	*P. esculentus*		
				LPeEs8	*P. esculentus*		
				LPeEs9	*P. esculentus*		
				LPeEs10	*P. esculentus*		
				LPeEs11	*P. esculentus*		
Veľký Lél - Lake	VLS	47.759833	17.944722	LSPeEs1	*P. esculentus*	Male	
				LSPeEs2	*P. esculentus*	Male	
				LSPeEs3	*P. esculentus*	Male	
				LSPeEs4	*P. esculentus*	Male	
				LSPeEs5	*P. esculentus*	Male	
				LSPeEs6	*P. esculentus*	Male	
				LSPeEs7	*P. esculentus*	Male	
				LSPeEs8	*P. esculentus*	Female	
				LSPeEs9	*P. esculentus*	Female	
				LSPeEs10	*P. esculentus*	Male	
				LSPeRi1	*P. ridibundus*	Female	
				LSPeRi2	*P. ridibundus*	Male	
				LSPeRi3	*P. ridibundus*	Male	
				LSPeRi4	*P. ridibundus*	Male	
				LSPeRi5	*P. ridibundus*	Male	
				LSPeRi6	*P. ridibundus*	Male	
				LSPeRi7	*P. ridibundus*	Male	
				LSPeRi8	*P. ridibundus*	Male	
				LSPeRi9	*P. ridibundus*	Male	
				LSPeRi10	*P. ridibundus*	Male	

Vinné	VI	48.816976	21.989034	58/23	*B. bufo*	Male	1
				59/23	*B. bufo*	Male	5
				60/23	*B. bufo*	Male	11
				61/23	*B. bufo*	Female	3
				62/23	*B. bufo*	Female	7
				63/23	*B. bufo*	Male	18
				64/23	*B. bufo*	Female	2
				65/23	*B. bufo*	Female	1
				66/23	*B. bufo*	Male	4
				67/23	*B. bufo*	Female	1
				68/23	*B. bufo*	Male	5
				69/23	*B. bufo*	Female	43
				70/23	*B. bufo*	Male	4
				71/23	*B. bufo*	Male	2
				72/23	*B. bufo*	Female	
				73/23	*B. bufo*	Female	2
				74/23	*B. bufo*	Male	3
				75/23	*B. bufo*	Male	7
				76/23	*B. bufo*	Male	3
				77/23	*B. bufo*	Male	
				78/23	*B. bufo*	Male	3
				79/23	*B. bufo*	Male	
				80/23	*B. bufo*	Male	3
				81/23	*B. bufo*	Female	3
				82/23	*B. bufo*	Male	2
				83/23	*B. bufo*	Male	8
				84/23	*B. bufo*	Male	
				85/23	*B. bufo*	Male	
				86/23	*B. bufo*	Male	
				87/23	*B. bufo*	Male	8
				88/23	*B. bufo*	Male	14
				89/23	*B. bufo*	Male	
				90/23	*B. bufo*	Male	21
				70/24	*B. bufo*	Male	28
				71/24	*B. bufo*	Male	
				72/24	*B. bufo*	Male	3
				73/24	*B. bufo*	Male	3
				74/24	*B. bufo*	Male	
				75/24	*B. bufo*	Male	11
				76/24	*B. bufo*	Male	
				77/24	*B. bufo*	Male	4
				78/24	*B. bufo*	Male	2
				79/24	*B. bufo*	Male	1
Vyšný Slivník	VS	49.112080	21.275219	148/24	*B. viridis*	Female	
				149/24	*B. viridis*	Female	
				150/24	*B. viridis*	Female	
				151/24	*B. viridis*	Female	
Zamarovce	ZA	48.909715	18.074896	105/23	*B. bufo*	Male	
				106/23	*B. bufo*	Male	6
				107/23	*B. bufo*	Male	13
				108/23	*B. bufo*	Male	2
				109/23	*B. bufo*	Male	5
				110/23	*B. bufo*	Male	
				111/23	*B. bufo*	Male	4
				112/23	*B. bufo*	Male	6
				113/23	*B. bufo*	Male	
				114/23	*B. bufo*	Female	15
				115/23	*B. bufo*	Male	3
				116/23	*B. bufo*	Female	4
				117/23	*B. bufo*	Male	4
Železná studnička	ZS	48.190172	17.082313	115/24	*B. bufo*		
				116/24	*B. bufo*		
				117/24	*B. bufo*		1
				118/24	*B. bufo*	Male	
				119/24	*B. bufo*	Male	16
				120/24	*B. bufo*		
				122/24	*B. bufo*		2
				123/24	*B. bufo*	Male	35
				124/24	*B. bufo*		16
				125/24	*B. bufo*		
				126/24	*B. bufo*	Male	30
				130/24	*B. bufo*		
				131/24	*B. bufo*		
